# Reorganization of the ancestral sex-determining regions during the evolution of trioecy in *Pleodorina starrii*

**DOI:** 10.1038/s42003-023-04949-1

**Published:** 2023-06-09

**Authors:** Kohei Takahashi, Shigekatsu Suzuki, Hiroko Kawai-Toyooka, Kayoko Yamamoto, Takashi Hamaji, Ryo Ootsuki, Haruyo Yamaguchi, Masanobu Kawachi, Tetsuya Higashiyama, Hisayoshi Nozaki

**Affiliations:** 1 Department of Biological Sciences, Graduate School of Science, The University of Tokyo, Hongo, Bunkyo-ku, Tokyo 113-0033 Japan; 2 Biodiversity Division, National Institute for Environmental Studies, Onogawa, Tsukuba, Ibaraki 305-8506 Japan; 3 Department of Frontier Bioscience, Hosei University, Kajino-cho, Koganei, Tokyo, 184-8584 Japan; 4 Department of Chemical and Biological Sciences, Faculty of Science, Japan Women’s University, Bunkyo-ku, Tokyo 112-8681 Japan; 5 Research and Development Initiative, Chuo University, Kasuga, Bunkyo-ku, Tokyo 112-8551 Japan; 6 Department of Natural Sciences, Faculty of Arts and Sciences, Komazawa University, Komazawa, Setagaya-ku, Tokyo 154-8525 Japan

**Keywords:** Evolutionary genetics, Genome

## Abstract

The coexistence of three sexual phenotypes (male, female and bisexual) in a single species, ‘trioecy’, is rarely found in diploid organisms such as flowering plants and invertebrates. However, trioecy in haploid organisms has only recently been reported in a green algal species, *Pleodorina starrii*. Here, we generated whole-genome data of the three sex phenotypes of *P. starrii* to reveal a reorganization of the ancestral sex-determining regions (SDRs) in the sex chromosomes: the male and bisexual phenotypes had the same “male SDR” with paralogous gene expansions of the male-determining gene *MID*, whereas the female phenotype had a “female SDR” with transposition of the female-specific gene *FUS1* to autosomal regions. Although the male and bisexual sex phenotypes had the identical male SDR and harbored autosomal *FUS1*, *MID* and *FUS1* expression during sexual reproduction differed between them. Thus, the coexistence of three sex phenotypes in *P. starrii* is possible.

## Abstract (web summary)

Whole genome sequencing data reveals three distinct sex phenotypes derived from ancestral sex chromosome reorganization in a green algae species.

## Introduction

How sexual reproduction occurs within a species, and how different mating systems have evolved, have been studied by evolutionary biologists since the time of Charles Darwin. Two basic mating systems, dioecy and hermaphrodite (cosexuality or monoecy), are generally recognized^1^. The former includes two separate sex phenotypes (male and female), whereas the latter has only a single sex phenotype that produces both male and female gametes. Transitions between these two systems are frequently recognized across haploid and diploid eukaryotes^1,2^. In addition to these mating systems, three intermediate or mixed systems in diploid species are known in which unisexual and bisexual individuals coexist, namely gynodioecy (unisexual female and bisexual), androdioecy (unisexual male and bisexual), and trioecy (unisexual male, unisexual female, and bisexual) (Supplementary Table 1). Trioecy is very rare across the tree of life, and the genetics of sex determination has only been dissected in a very small number of species, including the nematode *Auanema*^3,4^ and the flowering plant *Carica papaya*^5,6^.

In haploid algae and fungi, heterothallic and homothallic mating systems are known^7^. The heterothallic mating system has two sex phenotypes that are genetically different: the unisexual male forms only male gametes and the unisexual female only female gametes. The homothallic mating system has a single sex genotype that forms both male and female gametes and is self-compatible within a clone. However, mixed mating systems, like “trioecy”, had not been reported in haploid species until recently (Supplementary Table 2)^8^.

Sex is determined by sex-determining regions (SDRs) in the U (female) or V (male) haploid sex chromosomes in heterothallic species of haploid algae and bryophytes^9^. In SDRs, recombination is suppressed, and genome sequences differ between males and females. SDRs harbor gametologs (homologous genes shared between male and female SDRs) and sex-specific genes that are only present in either male or female SDR. In the volvocine green algae, SDRs have been identified in six heterothallic species based on comparative genome analyses^10–13^. SDRs of mating type minus (*MT*−) in isogamous species and males harbor the minus mating type-determining gene *MID*^10,14,15^. On the other hand, SDRs of mating type plus (*MT*+) in isogamous species and females generally harbor the plus gamete-specific membrane protein gene *FUS1*^11–13,16,17^. In addition to *MID* and *FUS1*, several sex-specific genes were identified in the volvocine lineage including *MTD1*, which is required for male gametogenesis^11,13,18^. Homologs of sex-specific genes such as *MTD1* and gametologs have often been found in autosomal regions of some heterothallic volvocine species^12^.

**Fig. 1:**
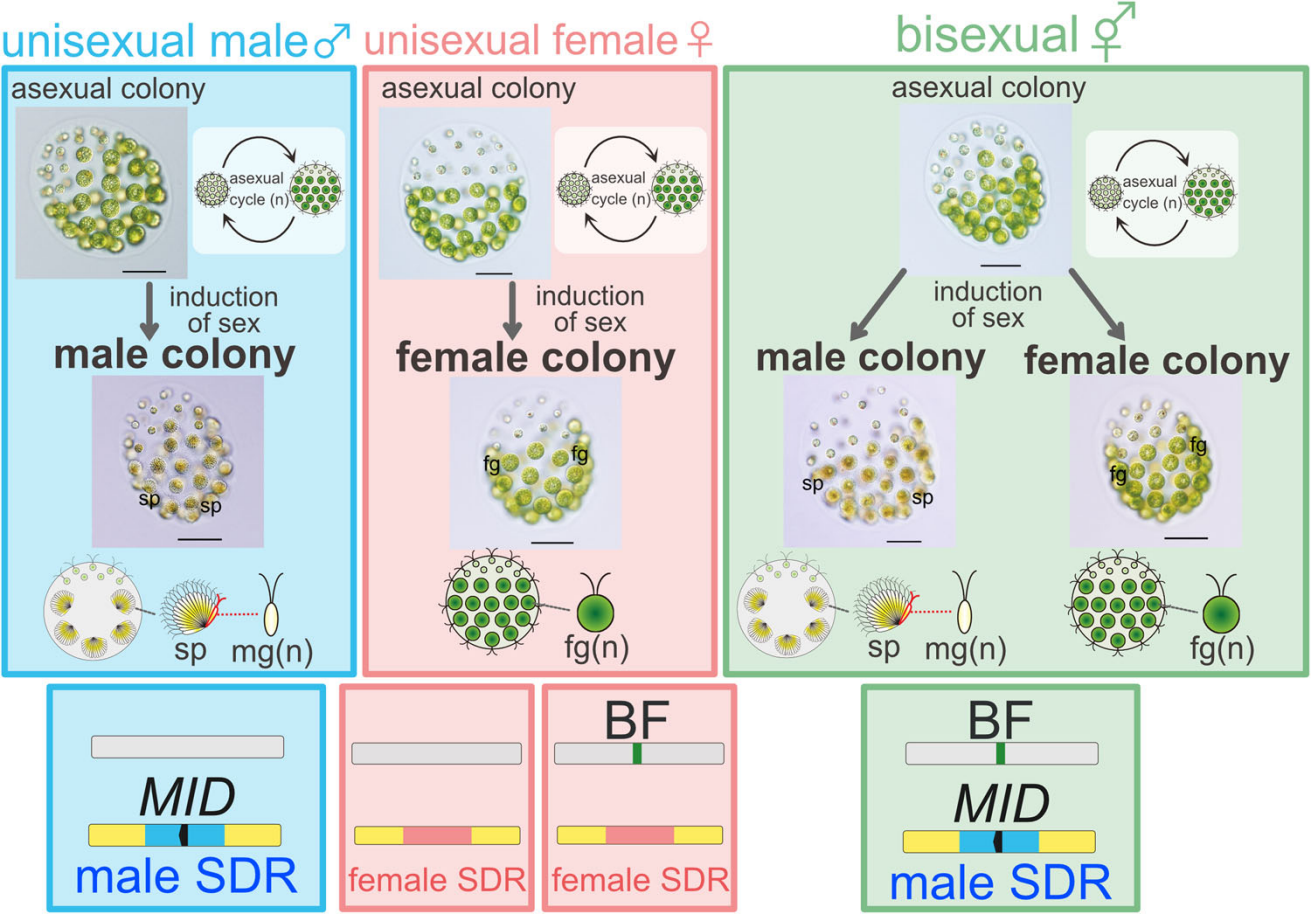
Schematic representation of three sex phenotypes and their possible genotypes, as deduced from intercrossing experiments and genomic polymerase chain reaction of *MID* in the haploid trioecious green alga *Pleodorina starrii* (based on Takahashi et al.^8^). Note that the unisexual male phenotype has a putative male sex-determining region (SDR, blue) in the V sex chromosome and lacks the bisexual factor (BF) in autosomes. The bisexual phenotype has a putative male SDR, but also has a BF in autosomes. The unisexual female phenotype has a putative female SDR (pink) in the U sex chromosome and harbors or lacks BF. Yellow and gray rectangles represent sex chromosomes and autosomes, respectively. sp sperm packet (bundle of male gametes), mg male gamete, fg female gamete. All scale bars = 50 µm.

We recently identified a haploid triecious species, the volvocine green alga *Pleodorina starrii*, from a Japanese water system that has three sex phenotypes: unisexual male (producing sexual male colonies that form only male gametes), unisexual female (producing sexual female colonies that form only female gametes), and bisexual (with both sexual male and female colonies) phenotypes (Fig. 1)^8^. Intercrossing experiments between unisexual and bisexual phenotypes of *P*. *starrii* culture strains revealed a possible genetic system that may determine the three sex phenotypes based on two independent loci: the putative SDR on the sex chromosome and the bisexual factor (BF) on the autosome (Fig. 1). The male and bisexual phenotypes have the identical “putative male SDR” whereas the female phenotype harbors the putative female SDR. BF plays a role in determining the bisexual phenotype in the presence of the putative male SDR (Fig. 1). This is a unique type of sex determination^19^. However, molecular genetic characteristics of the SDR and BF in *P. starrii* have not been elucidated.

The present study was undertaken to elucidate the molecular genetic basis of the haploid mating system with three sex phenotypes of *P. starrii* by generating de novo whole-genome sequences of the three sex phenotypes. Our comparative genomic analyses resolved unusual features of sex-related genes and sex chromosomes in *P. starrii*. The results suggested genomic basis for the coexistence of three sex phenotypes in a single haploid species and genomic reorganizations of the ancestral heterothallic sex determination system during the evolution of three sex phenotypes in *P. starrii*.

## Results

### Whole-genome assembly

We generated whole nuclear genomes of three sex phenotypes of *Pleodorina starrii* (Fig. 1) by assembling high-fidelity PacBio long sequencing reads into contigs (see Methods). After polishing the contigs with Illumina short reads, each genome was composed of 95–288 gap-free contigs (N50 = 0.83 to 3.12 Mbp). The total lengths were approximately 130 Mbp (Supplementary Table 3), consistent with the predicted genome sizes (Supplementary Fig. 1). The number of estimated protein coding genes supported by RNA sequencing data was approximately 17,000–18,500 (Supplementary Table 3). To evaluate genome annotations, we performed benchmarking universal single-copy orthologs (BUSCO) analysis (see Methods), which produced values of 97.3–98.2% (Supplementary Table 3). The completeness of the three whole-genome sequences was high based on the presence of most of the BUSCO reference genes (97.3–98.2% complete genes) (Supplementary Table 3). In addition, ploidy was estimated based on k-mer frequencies using GenomeScope^20^, and the k-mer histograms showed clear single peaks that unambiguously demonstrated haploidy among all three sex phenotypes (Supplementary Fig. 1).

### SDRs of the three sex phenotypes and sex-related genes

**Fig. 2:**
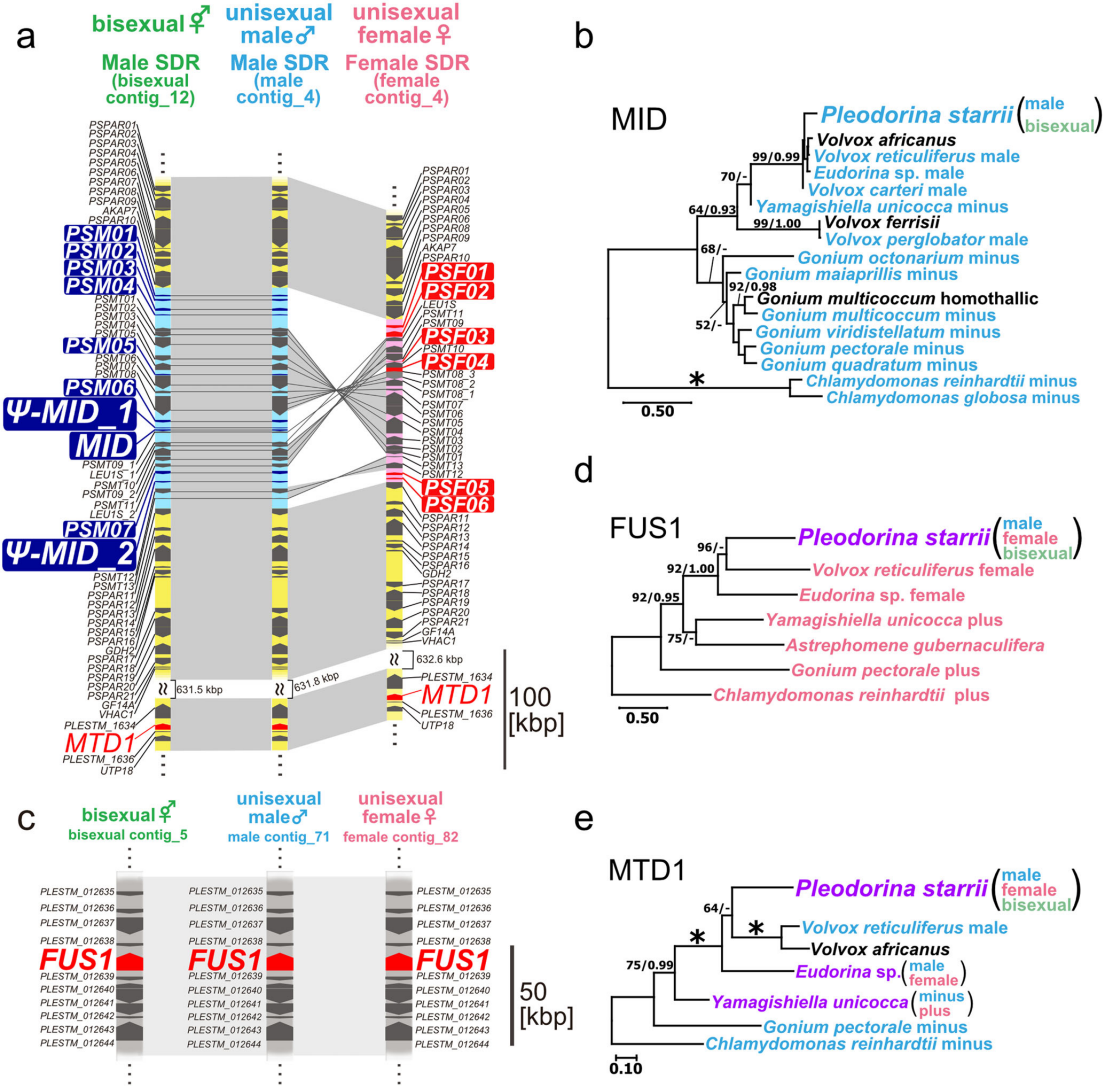
Sex-determining regions (SDRs) and homologs of sex-specific genes in the haploid trioecious green alga *Pleodorina starrii*. **a** Comparison of SDRs and adjacent regions of three sex phenotypes. Light blue and pink regions represent male and female SDRs, respectively. Genes with dark blue and red background represent male- and female-specific genes, respectively. Yellow regions represent pseudo-autosomal regions. Gray shading indicates a syntenic bloc. **b** Phylogeny of homologs of *MID* in the volvocine lineage, inferred based on 142 deduced amino acid sequences by the maximum likelihood (ML) method using the JTT + G model. Blue represents male- or mating type minus-specific genes. Black represents homologs of homothallic species. All positions containing gaps and missing data were eliminated from the alignment. Branch lengths are proportional to the evolutionary distances indicated by the scale bar. Numbers at left and right above branches indicate bootstrap values (BV) of the ML (≥50%) and posterior probabilities (PP) of Bayesian inference (≥0.90), respectively. Asterisks at the branches indicate 100% BV and 1.00 PP according to the two methods. **c** Comparison of autosomal regions harboring *FUS1* homologs of three sex phenotypes. Gray shading indicates a syntenic bloc. **d** Phylogeny of homologs of *FUS1* in the volvocine lineage, inferred based on 501 amino acid sequences deduced by ML using the LG + G + I model. Pink represents mating type plus-specific or female-specific genes within a SDR or possible SDR. Purple indicates autosomal genes harbored in all sex phenotypes of each species. For the others, see (**b**). **e** Phylogeny of homologs of *MTD1* in the volvocine lineage, inferred based on 495 amino acid sequences deduced by the ML method using the JTT + G model. Purple indicates autosomal genes harbored in all sex phenotypes of each species. For the others, see (**b**).

To examine the genetic bases of the three sex phenotypes of *P. starrii*, we examined their SDRs using the three whole genomes. Applying the basic local alignment search tool (BLAST N; National Center for Biotechnology Information) to *PlestMID*^8,15^ and male whole-genome data identified a contig (male contig_4) harboring *MID*. By comparing male contig_4 with the female whole genome data, we identified one contig (female contig_4) that had two separate regions almost identical to those of male contig_4. Dotplot analysis of male contig_4 and female contig_4 revealed male and female SDRs flanked by two regions [pseudo autosomal regions (PARs)] that were almost identical (Supplementary Fig. 2). The male and female SDRs were approximately 187 and 137 kbp long, respectively, and exhibited three inverted regions (measuring approximately half the SDR in total) (Fig. 2a; Supplementary Fig. 2). Comparative analysis of male contig_4, female contig_4, and the whole-genome data of the bisexual phenotype revealed a contig of the bisexual phenotype (bisexual contig_12) harboring an SDR flanked by PARs. Dotplot analysis of the bisexual_12 and male_4 contigs revealed that their SDR and PARs were identical (Fig. 2a; Supplementary Fig. 3). Therefore, the bisexual genotype has a male SDR with *PlestMID* (Fig. 2a, b), as suggested by previous intercrossing experiments^8^.

Male and female SDRs shared 14 gametologs (*PSMT01-13* and *LEU1S*). *PSMT09* and *LEU1S* were duplicated in the male SDR, whereas three paralogs of *PSMT08* were present in the female SDR. All these paralogs seemed functional genes. The male and female SDRs harbored 10 male-specific genes (*PSM01-07*, *MID*, *ψ-MID_1*, and *ψ-MID_2*) and six female-specific genes (*PSF01-06*), respectively. In contrast to other heterothallic volvocine species^10–13^, the female SDR lacked *FUS1*, and the male SDR contained three paralogs of *MID* (Supplementary Fig. 4). One of the three *MID* paralogs was functional and encoded the full MID protein sequence (163 amino acids) whereas the other two were pseudogenes encoding only 75 or 120 amino acids (Supplementary Fig. 4). Other male-specific genes lacked conserved putative domains except for *PSM03*, which has the FTP (eel-fucolectin fachylectin-4 pentaxrin-1) protein-protein interaction domain. BLASTP of these male-specific genes lacking conserved domains as queries [cutoff maximum e-value: 1e-10] showed their similarity to “hypothetical protein”. Among the six female-specific genes, a conserved domain was identified only in *PSF06* [PRK12678 domain (transcription termination factor Rho)]. Five other female-specific genes had no homologs according to BLASTP analysis [cutoff maximum e-value: 1e-10].

In addition, the divergence of gametologs between male and female SDRs was calculated using synonymous and non-synonymous substitutions (dS and dN, respectively) and compared to other volvocine species (Supplementary Fig. 5). Both dS and dN rates were low in *P*. *starrii* (<0.15). All dN/dS values (ω) in *P*. *starrii* were <1.0. Except for *LEU1S* (Supplementary Fig. 6), all gametologs in *P*. *starrii* lack homologous genes in other volvocine algae. Such gametologs in *P. starrii* cannot be aligned with homologous sequences from other volvocine green algae. Thus, the codon-based test for positive selection was only examined in *LEU1S*, suggesting no positive selection in *LEU1S* (Supplementary Table 4). Since the synonymous divergence between gametologs in *P. starrii* is quite low and the majority of the gametologs in *P. starrii* are localized within the inverted regions (Supplementary Figs. 2, 5), this suggests a recent origin of the *P. starrii* SDR or a recent gene conversion (inverted regions) between male and female SDRs.

Male SDRs had lower GC content, lower gene density, and a higher repeat rate than the total genome, which is characteristic of typical SDRs^10–13^. However, the GC content, gene density, and repeat rate were almost identical between female SDRs and the total genome (Supplementary Tables 3, 5).

Analysis of the three whole genomes revealed that each sex genotype had *FUS1* in the autosomal region, although *FUS1* is usually present in the female SDR in other volvocine species^11–13,21^. *FUS1* was present in male contig_71, female contig_82, and bisexual contig_5 (Fig. 2c, d). Dotplot comparisons showed that the sequences of the regions surrounding *FUS1* were almost identical among the three sex phenotypes (Supplementary Fig. 7). Reverse transcription polymerase chain reaction (RT-PCR) also revealed the exon-intron structure of *FUS1*, and the sequence of the coding region was identical among the three sex phenotypes (Supplementary Fig. 8). The amino acid sequence had one signal sequence, one transmembrane domain, and five immunoglobulin-like repeats, as in other volvocine species^12,13^ (Supplementary Fig. 9), suggesting that the *FUS1* ortholog of *P*. *starrii* may function as a gamete adhesion factor as in *Chlamydomonas reinhardtii*^17^.

We also examined two conserved sex-related genes, *MTD1*^12,13^ and *GCS1*/*HAP2*^22^, in *P*. *starrii*. *MTD1* is thought to regulate *MID* in *Chlamydomonas reinhardtii* and is found in several volvocine species. *MTD1* is male-specific (present in the male SDR) in the oogamous volvocine species *Volvox reticuliferus* or found in the PAR adjacent to the SDR in *Y*. *unicocca* and *Eudorina* sp^12,13^.

In all three sex phenotypes of *P*. *starrii*, *MTD1* was localized in the PAR (Fig. 2a; Supplementary Fig. 10). The deduced amino acid sequence of *P*. *starrii MTD1* showed a conserved exon-intron structure and multiple armadillo/beta-catenin-like repeat domains (Supplementary Figs. 11, 12), suggesting functional similarity among volvocine species. Phylogenetic analysis of *MTD1* suggested multiple transpositions of *MTD1* between SDR and PAR during volvocine evolution (Fig. 2e).

### Expansion of *GCS1/HAP2*

**Fig. 3:**
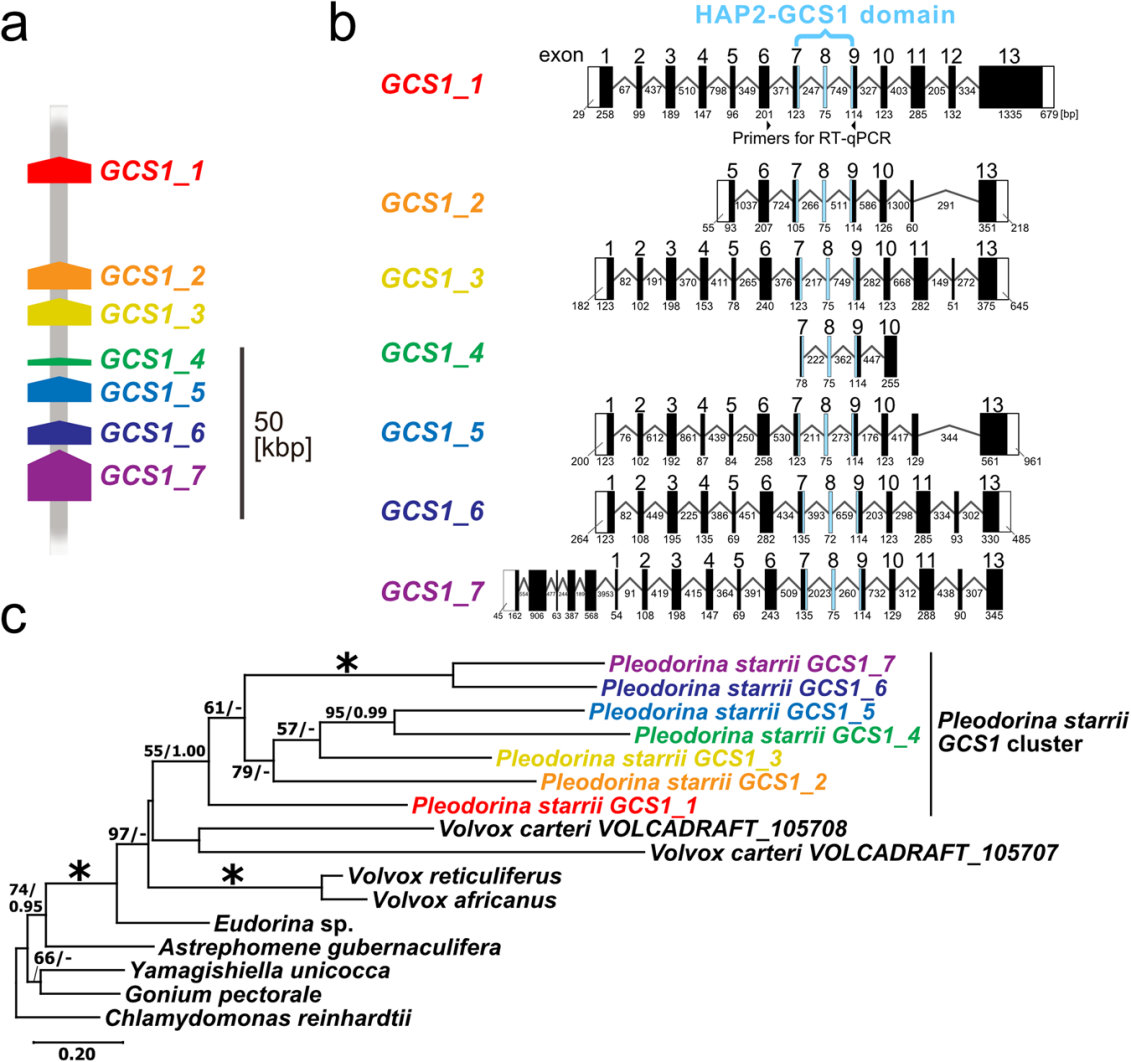
Seven *GCS1/HAP2* paralogs in *Pleodorina starrii*. **a** Diagram of the *GCS1*/*HAP2* cluster localized within a 100 kbp autosomal region of three sex phenotypes (Supplementary Fig. 13). **b** Exon-intron structures of the seven paralogs from unisexual male. Filled and open boxes represent exon sequences and non-coding (UTR) sequences, respectively. Lines between boxes indicate intron sequences. The number above the box indicates the homologous exon in *GCS1_1*. Numbers below boxes and lines indicate the numbers of nucleotides. Light blue regions represent the HAP2-GCS1 domain coding sequences. The positions of *GCS1_1* specific primers (Supplementary Table 10) used for reverse transcription quantitative PCR (RT-qPCR) analysis (Fig. 4) are also indicated. **c** Phylogeny of seven paralogs of *P*. *starrii* in the volvocine lineage, inferred from amino acid sequences of *GCS1* (698 amino acids, see Supplementary Fig. 15) deduced by the maximum likelihood (ML) method using the WAG + G + I model. All aligned positions were used for ML analysis (MEGAX option, Gaps/Missing Data Treatment: Use all sites). Branch lengths are proportional to the evolutionary distances indicated by the scale bar. Numbers at left and right above branches indicate bootstrap values (BV) of the ML (≥50%) and posterior probabilities (PP) of Bayesian inference (≥0.90), respectively. Asterisks at the branches indicate 100% BV and 1.00 PP according to the two methods.

*GCS1*/*HAP2* is a single transmembrane gamete fusion factor that contains HAP2-GCS1 and functions specifically in isogamous *MT*- and males^22^, although *GCS1* homologs are localized in autosomal regions of volvocine algae^10–13^. Interestingly, *GCS1* in *P*. *starrii* was expanded to represent seven paralogs clustered within an approximately 100-kbp genome sequence in autosomal regions in all three phenotypes (Fig. 3a, b). Furthermore, the cluster was duplicated and found in two different contigs in the bisexual genotype (bisexual contig_1 and contig_7) (Supplementary Fig. 13). However, whether such a duplication was present or absent was ambiguous in male and female genomes due to short contigs harboring the cluster in the male and female genome data sets and almost completely identical sequences between the two *GCS1* clusters in the bisexual genome (Supplementary Fig. 13). Dotplot analysis showed that the approximately 130-kbp genome sequence containing the *GCS1* cluster was nearly identical among the three sex phenotypes (Supplementary Fig. 13b). Each cluster was composed of seven tandemly arranged paralogs and each paralog had a conserved HAP2-GCS1 domain, whereas non-domain sequences and exon-intron structures were diversified between paralogs within the cluster (Fig. 3b; Supplementary Figs. 14, 15). Phylogenetic analysis suggested that all seven paralogs constituted a monophyletic group, suggesting their diversification or expansion after the origin of *P*. *starrii* or its recent ancestor (Fig. 3c). More genome data for *GCS1* in volvocine algae closely related to *P*. *starrii* are needed to fully elucidate the origin of the seven paralogs in *P*. *starrii*.

### Expression of sex-related genes in the three sex phenotypes

**Fig. 4:**
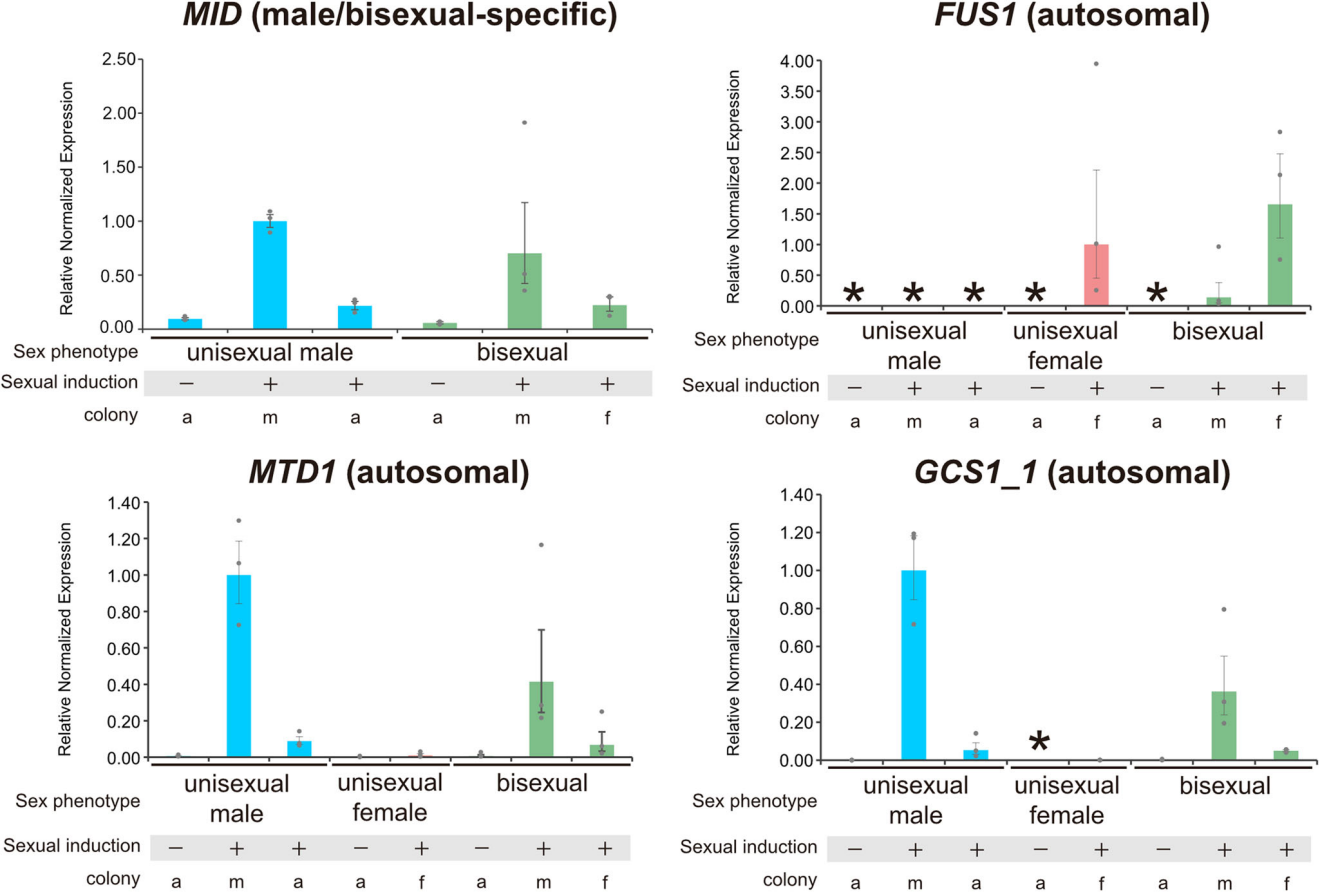
Reverse transcription quantitative PCR analysis of four sex-related genes (*MID*, *FUS1*, *MTD1*, and *GCS1_1*) in *Pleodorina starrii*, based on three biological replicates (dots) for all samples. Normalized expression of four sex-related genes in *P*. *starrii* asexual and sexual colonies relative to the expression of the unisexual male sexual colony (male colony) for *MID*, *MTD1*, and *GCS1_1* and unisexual female sexual colony (female colony) for *FUS1*. Bars show standard error of the mean (SEM). *, no expression detected; –, not sexually induced; +, sexually induced; a, asexual colony; m, male colony; f, female colony. For the original numerical data, see Supplementary Data 2.

To investigate the expression of the four sex-related genes *MID*, *FUS1*, *MTD1*, and *GCS1* in the three sex phenotypes, reverse transcription quantitative PCR (RT-qPCR) analyses were performed using asexual and sexual colonies in the three sex phenotypes (see Methods). The expression of *MID* was upregulated in male colonies and asexual colonies, in unisexual male and bisexual genotypes under sexually induced conditions, compared to asexual colonies cultured under sexually uninduced conditions (Fig. 4). The upregulation of *MID* was greater in male colonies of unisexual male and bisexual genotypes compared with asexual colonies under the same sexually induced conditions. The expression of *MTD1* and *GCS1* was upregulated in male colonies of unisexual male and bisexual genotypes, but not in the unisexual female genotype (Fig. 4). Thus, although these two genes were present in the autosomal region and harbored the three sex phenotypes, expression may be related to male gametes (*MT*− in isogamous species), as in other volvocine species^12,18,22^.

As shown by our whole-genome data, *P*. *starrii* is unique in that *FUS1*, a female-specific gene conserved in other volvocine species, was present in an autosomal region in the three sex phenotypes. However, the expression of *FUS1* in *P*. *starrii* was strongly suppressed in the male genotype even when the culture was sexually induced. In bisexual and female genotypes, *FUS1* expression was upregulated in sexually induced female colonies. These results suggested a difference in the regulation of *FUS1* expression between the bisexual and male phenotypes. In *Chlamydomonas reinhardtii*, transformation of *MID* into the *MT*+ genotype (with *FUS1*) results in strong suppression of *FUS1*^14^. However, both bisexual and male genomes of *P*. *starrii* harbor *MID* and *FUS1*. Therefore, the male genotype of *P*. *starrii* may reflect the same gene regulation between *MID* and *FUS1* as in the *MID*-transformed *MT*+ of *C*. *reinhardtii*. In the bisexual genotype of *P*. *starrii*, however, this regulation or suppression of *FUS1* by *MID* may be modified by an unknown factor specific to the bisexual genotype. Even under the same sexually induced conditions of the bisexual genotype, the expression of *MID* and *FUS1* was different between sexual male and sexual female colonies. Therefore, expression of the sex-related genes is not sex genotype-specific; rather, it represents male or female colony specificity; the genes are critically regulated in the bisexual phenotype to produce both male and female gametes in a single haploid genotype.

## Discussion

In the heterothallic volvocine species, male and female phenotypes are determined by the presence of male and female SDRs, respectively, and each SDR harbors a sex-specific gene that plays critical roles in the formation and function of male or female gametes; this gene is *MID* in the male SDR and *FUS1* in the female SDR^10–13^. Therefore, the formation of both male and female gametes is not permitted in each sex genotype (with male or female SDRs) of the heterothallic species. By contrast, our genome data clearly demonstrated that the haploid genome of *P*. *starrii* has the male SDR with *MID* or female SDR without *FUS1*, while *FUS1* is positioned in the autosomal region (Fig. 2). These fundamental genomic characteristics may allow the *P*. *starrii* haploid genotype to produce both male and female gametes within the genotype with the male SDR. Thus, molecular genetic or genomic bases for the three sex phenotypes in the single haploid species *P*. *starrii* might have principally been established by the transposition of *FUS1* from the ancestral female SDR to the autosomal region to localize both male SDR (with *MID*) and *FUS1* in a single haploid genome of male and bisexual genotypes.

Although the present study demonstrated that male and bisexual genotypes of *P. starrii* have both *MID* and *FUS1* (Fig. 2a, c), the expression of these sex-related genes differs at the colony level (Fig. 4). Thus, different regulatory mechanisms of *MID* and *FUS1* must have evolved between male and bisexual phenotypes during the origin of trioecy of *P*. *starrii*. This difference of transcriptional regulation may be caused by the presence or absence of BF, which is predicted to exist in the autosomal region of the bisexual phenotype based on the previous intercrossing experiments^8^ (Fig. 1). Thus, the sex-determination in *P. starrii* is probably based on the modification of the typical heterothallic system with male and female SDRs by the acquisition of two autosomal sex genes, *FUS1* and “BF” (Fig. 1). This situation is similar to the polygenic or multifactorial sex determination in the house fly *Musca domestica*^23–25^. *M. domestica* has X and Y sex chromosomes. In the ancestral state, the sex was determined by the presence or absence of a male-determining locus (M factor) on the Y chromosome^25^. Thus, typical males are XY^M^ and females are XX. However, males with the M factor on the autosomes (A^M^) or the X chromosome (X^M^) can be found in natural populations^25^. The stable frequency of A^M^ found in natural populations of *M. domestica* over 30 years suggests that the polygenic sex determination system of the house fly is not in transition^25^. Long-term field data on the three sex phenotypes of *P. starrii* are expected to determine whether or not the trioecy represents is a transient state.

Other unusual genomic features of *P*. *starrii* were found in this study. First, there were multiple or expanded paralogs of *MID* and *GCS1*. Although two paralogs of *GCS1* were found in *Volvox carteri*^26^, tandemly repeated *GCS1* paralogs within the cluster have not previously been reported in eukaryotic genomes. In all six heterothallic volvocine species previously examined by analyzing whole-genome data, only a single *MID* ortholog is present in the male (or *MT*- in isogamous species) SDR. However, three paralogs of *MID* were identified in the male SDR of *P*. *starrii*, two of which are pseudogenes (Fig. 2a). The homothallic volvocine species *Volvox africanus* has a cluster of five tandemly arranged *MID* paralogs, but all five of these have identical sequences encompassing the full coding regions^13^. Pseudogenes, which arise primarily from gene duplication, have a variety of functions including regulation of cognate gene transcription by antisense RNA^27^ and the production of small interfering RNA^28^. Such mechanisms may play a role in the differential transcriptional regulation of *MID*, *GCS1*, and their downstream sex-related genes among the three sex phenotypes in *P*. *starrii* (Fig. 1). Thus, paralogous expansion of *MID* and *GCS1* may have underpinned the origin of trioecy in *P. starrii*. Second, the female SDR did not exhibit the characteristics shared by heterothallic volvocine SDRs^13^, such as GC content and repeat rates (Supplementary Tables 3, 5), as discussed above. The transposition of *FUS1* from the ancestral female SDR to the autosomal region might have modified the genome characteristics of the female SDR, but the reasons for the unusual female SDR in *P*. *starrii* are unknown. However, one could only speculate that during the evolution of trioecy, the female SDR may have lost the processes by which SDRs in haploid systems tend to evolve differently from the rest of the genome^9^.

Homothallic species may have repeatedly evolved from heterothallic ancestors among volvocine algae^29^. Based on the recent phylotranscriptomic analysis of volvocine algae^30^, trioecious *P*. *starrii* and heterothallic *P*. *indica* constitute a small clade that belongs to a large monophyletic group (Pst-Vpow group) with two homothallic culture strains of *Eudorina elegans* (NIES-458 and NIES-568)^31^, homothallic *Volvox powersii*, heterothallic *V*. *gigas*, and *E*. *elegans* strain FACHB-2321 of unknown sexuality^32^ (Supplementary Fig. 16). Therefore, transition from a heterothallic to homothallic mating system might have occurred at least once during the evolution of the Pst-Vpow group. The present comparative genome study clearly demonstrated that the trioecious mating system in *P*. *starrii* was established based on fundamentally distinct genomic characteristics (e.g., autosomal *FUS1*, expanded *MID*, and unusual female SDR). Thus, the *P*. *starrii* trioecy may not be a slightly modified version of the mating system of the usual heterothallism found in the volvocine algae; rather, it may have evolved based on reorganization of the genome of ancestral heterothallic species before the divergence of *P*. *starrii* within the Pst-Vpow group. Thus, further comparative genomics of volvocine species closely related to *P*. *starrii* may resolve the common genomic bases for the evolution of trioecy and homothallism within the Pst-Vpow group.

Our study resolved unusual genomic features of the trioecious species *Pleodrina starrii*, including the transposition of *FUS1* from the female SDR to the autosomal region, paralogous expansions of *MID* and *GCS1*, and the low GC and low repeat rich female *SDR*. As discussed above, the transposition of *FUS1* to the autosomal region of the three sex phenotypes is the fundamental genetic basis for harboring the female gene *FUS1* and the male gene *MID* in a single haploid genome of the bisexual phenotype. However, the specific role of other genomic features in the evolution and maintenance of trioecy is unknown. In addition, our previous genetic analysis suggested a putative factor (BF) that determines the bisexual phenotype in the presence of the male SDR (Fig. 1). One could speculate that only one of these is actually sufficient, and that all the others are just neutral changes that have accumulated later without functional consequences for the mating system.

## Methods

### Algal culture strains

For whole-genome sequencing of the three sex phenotypes of *Pleodorina starrii*, NIES-1363 (unisexual male^15,33^), NIES-4481 [= “2P1”, unisexual female (without BF based on crossing experiments with a unisexual male)^8^], and NIES-4479 (= “P85”, bisexual^8^) were used. Due to the long-term maintenance of cultures, the inducibility of sexual reproduction has decreased in NIES-1363 and NIES-4479. Thus, alternative sexually active strains, i.e., NIES-4480 (= “P7”, unisexual male^8^) and NIES-4482 (= “P10”, bisexual^8^), were used for RNA sequencing and gene expression analyses (see below). All strains were kept at 20 °C in screw-cap tubes containing 10 mL AF-6 medium^31,34^, under a 14-h light:10-h dark schedule with cool-white fluorescent lamps at an intensity of 55–80 μmol·m^−2^·s^−1^.

### Asexual and sexually induced samples of *P*. *starrii*

To prepare asexual samples of the three sex phenotypes of *P*. *starrii*, 0.25 mL of an actively growing culture of each sex phenotype in AF-6 medium was transferred to 10 mL of new VTAC medium^31,35^ and kept at 25 °C under a 14-h light:10-h dark schedule with cool-white fluorescent lamps at an intensity of 180–220 μmol·m^−2^·s^−1^ for 3 days. Sexually induced samples were prepared as described by Takahashi et al.^8^ with some modifications: 3-day-old culture grown in 10 mL VTAC + soil medium^8^ was mixed with 20 mL mating medium^35^ and 10 mL condition medium [fluid of sexually induced unisexual male culture filtered through a Millex-GP Filter (0.22 µm, PES = 33 mm, non-sterile; Merck-Sigma/Aldrich, Burlington, MA, USA)] in Petri dishes (20 × 90 mm) and grown at 25 °C under a 14-h light:10-h dark schedule with cool-white fluorescent lamps at an intensity of 180–220 μmol·m^−2^·s^−1^. Sexual colonies developed within 1 (unisexual male) or 2 days (unisexual female and bisexual).

### Genomic DNA sequencing and de novo whole genome assembly

Asexual samples of three sexual phenotypes of *P*. *starrii* were prepared as described above. Approximately 300 mL of culture of a single phenotype was centrifuged at 3500 rpm for 20 min using a TOMY LC-100 swing bucket rotor (TOMY DIGITAL BIOLOGY CO., LTD., Tokyo, Japan) to produce a pellet or concentrated cells or colonies. The pellet was frozen in liquid nitrogen and crushed with a dispensing spoon until the colonies were thawed. After thawing, Proteinase K and H1 lysis buffer from the NucleoBond^TM^ High Molecular Weight DNA kit (MACHEREY-NAGEL GmbH & Co. KG, Düren, Germany) were added to the crushed cells. Each sample was incubated in a water bath at 50 °C for 18 h. Subsequent procedures were performed according to the kit manufacturer protocols to prepare genomic DNA samples for whole-genome sequencing.

Genomic DNA samples from the three sex phenotypes were sent to Rhelixa (Tokyo, Japan) for long read sequencing using the by PacBio Sequel II instrument (Pacific Biosciences, Menlo Park, CA, USA) and SMRTbell® Express Template Prep Kit 2.0 (Pacific Biosciences), along with short read sequencing using the Illumina NovaSeq 6000 instrument (150 bp × 2 library; Illumina, San Diego, CA, USA) with the NEBNEXT® Ultra DNA Library Prep Kit for Illumina (New England Biolabs, Ipswich, MA, USA). Raw reads derived from the analyses using the PacBio and Illumina instruments were obtained from Rhelixa; their details are summarized in Supplementary Table 6.

For NIES-1363 (unisexual male) and NIES-4481 (unisexual female), the PacBio reads were assembled using Raven software (version 1.6.0)^36^. The assembly was polished using pbmm2 (version 1.8.0; Pacific Biosciences) and gcpp (version 2.0.2; Pacific Biosciences) with the default options. The Illumina reads were trimmed using fastp (version 0.20.1)^37^. Subsequently, the PacBio assemblies were polished using NextPolish (version 1.4.0)^38^ with the trimmed Illumina reads.

For NIES-4479 (bisexual), the PacBio reads were assembled using flye (version 2.8.3)^39^ and NextDenovo (version 2.4.0)^40^. The trimming of Illumina reads and polishing of the two PacBio assemblies were performed as described in NIES-1363 and NIES-4481. The two assemblies were merged using quickmerge (version 0.3)^41^ with the NextDenovo contigs as the query sequence. For the PacBio and trimmed Illumina reads, gaps were filled using TGS-GapCloser (version 1.1.1)^42^ and pilon 1.2.3^43^.

Putative duplicated contigs and contigs with irregularly high- or low-read coverage were removed using Purge Haplotigs (version 1.1.1)^44^ with default options. Bacterial contamination was checked using checkM (version 1.1.3)^45^, and putative bacterial contigs were removed. Organellar genomes were identified and removed using BLASTN against available chloroplast (JX977846.1) and mitochondrial (JX977845.1) genomes of *P*. *starrii*. Repeats were identified and soft-masked using RepeatModeler (version 2.0.2) and RepeatMasker (version 4.1.2)^46^. Finally, the whole-genome assemblies of NIES-1363 (unisexual male), NIES-4481 (unisexual female), and NIES-4479 (bisexual) were composed of 242 (134.8 Mbp, N50 = 1.06 Mbp), 288 (135.0 Mbp, N50 = 0.83 Mbp), and 95 contigs (136.1 Mbp, N50 = 3.1 Mbp), respectively. To check genome completeness, BUSCO analyses were performed using BUSCO (version 5.0.0)^47^ and the chlorophyta_obd10 dataset with the “genome mode” option selected; the completeness values were 98.2% (NIES-1363), 97.3% (NIES-4481), and 97.5% (NIES-4479).

### RNA-seq data

Asexual and sexually induced samples for the three sexual phenotypes of *P. starrii* were prepared and collected as described above. Total RNAs were extracted using the RNeasy Plant Mini Kit (Qiagen, Venlo, Netherlands), and treated with DNase I (amplification grade; ThermoFisher Scientific, Waltham, MA, USA). The total RNAs were sent to Rhelixa. Only mRNAs were isolated from samples using NEBNEXT® Poly(A) mRNA Magnetic Isolation Module (New England Biolabs) and Illumina paired-end libraries were constructed using NEBNext® Ultra^TM^ II Directional RNA Library Prep Kit (New England Biolabs). The constructed libraries were sequenced using the Illumina NovaSeq 6000 instrument (150 bp × 2 library). Raw reads were sent from Rhelixa; their details are summarized in Supplementary Table 7.

### Annotation of assembled whole genomes

Assembled whole-genome sequences and RNA sequencing reads of both asexual and sexually induced samples, obtained as described above, were used for annotation by Funannotate (version 1.8.9)^48^. To evaluate the completeness of the annotated gene set, protein-mode BUSCO analyses were performed using BUSCO (version 5.3.2)^47^ and the chlorophyta_odb10 dataset, with the “protein mode” option selected; the completeness values were 98.9% (unisexual male, NIES-1363), 97.5% (unisexual female, NIES-4481), and 97.5% (bisexual, NIES-4479).

### SDR identification

Candidate contigs harboring the entire SDR in three sex phenotypes (unisexual male, unisexual female and bisexual) were screened. First, a unisexual male contig with *MID* (male contig_4) was identified by BLASTN searches using BLAST+ v2.12.0 local blast^49^ against the present genome assembly of unisexual male with the *PlestMID* sequence (AB272616^15^) as the queries (cutoff maximum E-value: 1e-10). Next, BLASTN searches using BLAST+ were performed against the genome assemblies of unisexual female and bisexual with male contig_4 as the query (cutoff maximum E-value: 1e-10). “Female contig_4” was identified within the unisexual female assembly having two separate genome regions [pseudo autosomal regions (PARs)] homologous to those of male contig_4, while bisexual contig_12 had the male SDR (see below) flanked by two PARs. Dotplot analyses were carried out between male contig_4, female contig_4, and bisexual contig_12 using YASS^50^ to detect the rearranged genomic regions or SDR.

### Sex-related genes identification

TBLASTN searches using BLAST+ were performed against the genome assemblies of *P. starrii* unisexual male, unisexual female, and bisexual phenotypes with the volvocine sex-related proteins (Supplementary Table 8) as the queries (cutoff maximum E-value: 1e-10), and retrieved sequences with the highest similarity. For *FUS1* homologs of *P. starrii*, coding regions were determined by using reverse transcription polymerase chain reaction (RT-PCR). The polyadenylated mRNAs were directly isolated from sexually induced cultures of *P. starrii* unisexual female and bisexual strains using Dynabeads Oligo(dT)_25_ (Thermo Fisher Scientific), then mRNAs were separated from beads by incubation in e-HeatingBucket EHB (TAITEC CORPORATION, Saitama, Japan) at 65 °C for 5 min. Isolated mRNAs were reverse transcribed with the mixture of Oligo (dT)_20_ (Thermo Fisher Scientific) and random hexamers (Thermo Fisher Scientific) by Superscript III reverse transcriptase (Thermo Fisher Scientific), and subjected to PCR (2 min at 94 °C, followed by 35 cycles of 10 sec at 98 °C and 30 sec at 68 °C) with specific primers (Supplementary Table 9) and KOD One (TOYOBO, Osaka, Japan). To extend the cDNA sequences, 5’ RACE and 3’ RACE were performed using the GeneRacer Kit (Thermo Fisher Scientific) with specific primers (Supplementary Table 9). The PCR products were directly sequenced using an ABI PRISM 3100 Genetic Analyzer (Applied Biosystems, Foster City, CA, USA) with a BigDye^TM^ Terminator v3.1 Cycle Sequencing Kit (Thermo Fisher Scientific). Based on the cDNA sequence and genome sequence, the exon-intron structure of *FUS1* was determined.

### Molecular phylogenetic analyses of SDR genes and sex-related genes

Phylogenetic analyses were performed using MUSCLE^51^-aligned full-length protein sequences of SDR genes (sex-specific genes plus gametologs) and sex-related genes (Supplementary Table 8). Except for GCS1/HAP2, the maximum likelihood (ML) method was subjected to each alignment with complete deletion option and bootstrap values^52^ based on 1000 replications by MEGA X^53^ using the best-fitted model selected by MEGA X. In addition, Bayesian inference (BI) for the respective alignments was carried out using MrBayes v3.2.7a^54^ with the best-fitted model selected by Modeltest-NG v0.1.643^55^. Convergences of Markov chain Monte Carlo iterations were evaluated based on the average standard deviation of split frequencies for every 1,000,000 generations, discarding the first 25% as burn-in, and the iterations were automatically stopped when the average standard deviations were below 0.01, indicating convergence. For the GCS1/HAP2 alignment including seven paralogs of *P. starrii* (Fig. 3), amino acid sequences corresponding to 17-649 positions (including only outer domain sequences) of *CrGCS1* (XP_001695893) were subjected to ML and BI as described above except for using with “Use all sites” option in ML analysis.

The alignments used for the phylogenetic analyses are available in TreeBASE (Study ID: S29920)^56^. List of accession numbers used in the phylogenetic analysis is shown in Supplementary Table 8.

### Molecular evolutionary analysis

Divergence scores of DS and DN of gametologs between male and female SDRs (Supplementary Data 1) were computed using yn00 of the PAML4 package^57^; DN and DS site divergence of aligned coding sequences of gametologs was calculated based on Yang and Nielsen^58^ with equal weighting between pathways, and the same codon frequency for all pairs^11^.

### RT-qPCR of sex-related genes

From asexual samples of the three sex phenotypes, 50 asexual colonies were collected from each culture using a micropipette. Within sexually induced samples of unisexual male and female strains, sexual male and female colonies, respectively, developed and 50 of the sexual colonies were collected from each sample. Bisexual strain produced both male and female sexual colonies in the same sexually induced sample, from which 50 colonies of each sexual type were collected. Although sexual female colonies are morphologically indistinguishable from mature asexual colonies in *P. starrii*^33^ (Fig. 1), we collected relatively matured colonies without sperm packet formation and embryogenesis as “sexual female colonies” in sexually induced cultures of unisexual female and bisexual strains. In order to examine the difference between colonies during sperm packet formation (male colonies) and asexual colonies within the same sexually induced culture of unisexual male strain, 50 relatively mature asexual colonies were also collected and examined. We prepared three biological replicates for all samples. The polyadenylated mRNAs were isolated using Dynabeads Oligo (dT)_25_ and reverse transcribed as described above. Purified cDNAs were dissolved in 10 mM Tris-HCl. Gene specific primers were designed in a manner that the amplicons cross one or more exon-exon junctions. Standard curves were drawn with candidate primer pairs using cDNA sample of sexually induced male colony from unisexual male for *MID*, *MTD1*, *GCS1* and cDNA sample of sexually induced female colony from unisexual female for *FUS1*. Primer pairs with reaction efficiency closest to 100% were used for analyses: EF1L_qF2-EF1L_qR2 for *EF-1 like* (98.9%); MID_qF2-MID_qR2 for *MID* (102.3%); FUS1_qF3-FUS1-qR3 for *FUS1* (102.4%); MTD1_qF3-MTD1_qR4 for *MTD1* (98.7%); GCS1_qF2-GCS1_qR2 for *GCS1_1* (107.9%) (*SI Appendix*, Supplementary Table S10). qPCR was performed by using TB Green® Premix Ex Taq^TM^ II (Takara Bio Inc., Shiga, Japan) and Thermal Cycler Dice® Real Time System II (Takara Bio Inc.) with three technical replicates per one biological replicate and calculated mean Cq. The values of relative normalized expression (Supplementary Data 2) were computed using the *EF-1 like* gene as the reference gene based on Taylor et al.^59^.

### Oligo sequences of primers

Nucleotide sequences of all primers used in the present study are described in Supplementary Tables 9, 10.

### Statistics and reproducibility

Phylogenetic relationships within the phylogenetic trees constructed in the present study were tested with bootstrap values^52^ based on 1000 replications by MEGA X^53^ and posterior probabilities by Bayesian inference (BI) using MrBayes v3.2.7a^54^ with the best-fitted model selected by Modeltest-NG v0.1.643^55^. For BI, convergences of Markov chain Monte Carlo iterations were evaluated based on the average standard deviation of split frequencies for every 1,000,000 generations, discarding the first 25% as burn-in, and the iterations were automatically stopped when the average standard deviations were below 0.01, indicating convergence. To analyze and draw the figures of non-synonymous and synonymous substitutions of gametologs (Supplementary Data 1) and relative normalized expressions of RT-qPCR analysis (Supplementary Data 2), we used Microsoft® Excel® 2016. In the RT-qPCR analysis of sex-related genes, three biological replicates were examined for all samples, and the average relative normalized and log transformed expression for each biological replicate were calculated using the GEOMEAN function. The standard deviations were calculated using the STDEV function.

### Reporting summary

Further information on research design is available in the Nature Portfolio Reporting Summary linked to this article.

## Supplementary information

Peer Review File

Supplementary Information

Description of Additional Supplementary Files

Supplementary Data 1

Supplementary Data 2

Reporting Summary

## Data Availability

Raw reads of genome and RNA-seq data and genome assemblies with annotations data have been deposited in DNA Data Bank of Japan (DDBJ)^60^/European Molecular Biology Laboratory (EMBL)/GenBank (DDBJ Sequence Read Archive [DRA]: DRA015329; whole-genome assemblies with annotations: BRXT01000001through BRXT01000242 [*P. starrii* NIES-1363 male phenotype], BRXV01000001 through BRXV01000288 [*P. starrii* NIES-4481 female phenotype], and BRXU01000001 through BRXU01000095 [*P. starrii* NIES-4479 bisexual phenotype]]). SDR genome sequences harboring rearranged domains with sex-specific genes in *P. starrii* male, female and bisexual phenotypes, are available under accession numbers LC740503 through LC740521. Source data underlying Supplementary Fig. 5 and Fig. 4 are presented in Supplementary Data 1 and 2, respectively.

## References

[1] Weeks SC (2012). The role of androdioecy and gynodioecy in mediating evolutionary transitions between dioecy and hermaphroditism in the animalia. Evolution.

[2] Pannell JR (2002). The evolution and maintenance of androdioecy. Annu. Rev. Ecol. Syst..

[3] Félix MA (2004). Alternative morphs and plasticity of vulval development in a rhabditid nematode species. Dev. Genes Evol..

[4] Pires-daSilva A (2007). Evolution of the control of sexual identity in nematodes. Semin. Cell. Dev. Biol..

[5] Wang J (2012). Sequencing papaya X and Y h chromosomes reveals molecular basis of incipient sex chromosome evolution. Proc. Natl Acad. Sci. USA.

[6] Ueno H (2015). Genome sequence comparison reveals a candidate gene involved in male–hermaphrodite differentiation in papaya (*Carica papaya*) trees. Mol. Genet. Genom..

[7] Bold, H. C., Alexopoulos, C. J. & Delevoryas, T. Morphology of Plants and Fungi. 5th Edn. Harper & Row, New York (1987).

[8] Takahashi K (2021). Three sex phenotypes in a haploid algal species give insights into the evolutionary transition to a self-compatible mating system. Evolution.

[9] Coelho SM, Gueno J, Lipinska AP, Cock JM, Umen JG (2018). UV chromosomes and haploid sexual systems. Trends Plant Sci..

[10] Ferris P (2010). Evolution of an expanded sex-determining locus in *Volvox*. Science.

[11] Hamaji T (2016). Sequence of the *Gonium pectorale* mating locus reveals a complex and dynamic history of changes in volvocine algal mating haplotypes. G3 (Bethesda).

[12] Hamaji T (2018). Anisogamy evolved with a reduced sex-determining region in volvocine green algae. Commun. Biol..

[13] Yamamoto K (2021). Three genomes in the algal genus *Volvox* reveal the fate of a haploid sex-determining region after a transition to homothallism. Proc. Natl Acad. Sci. USA.

[14] Ferris PJ, Goodenough UW (1997). Mating type in *Chlamydomonas* is specified by *mid*, the *minus*-dominance gene. Genetics.

[15] Nozaki H, Mori T, Misumi O, Matsunaga S, Kuroiwa T (2006). Males evolved from the dominant isogametic mating type. Curr. Biol..

[16] Ferris PJ, Woessner JP, Goodenough UW (1996). A sex recognition glycoprotein is encoded by the plus mating-type gene *fus1* of *Chlamydomonas reinhardtii*. Mol. Biol. Cell.

[17] Misamore MJ, Gupta S, Snell WJ (2002). The *Chlamydomonas fus1* protein is present on the mating type plus fusion organelle and required for a critical membrane adhesion event during fusion with minus gametes. Mol. Biol. Cell.

[18] Lin H, Goodenough UW (2007). Gametogenesis in the *Chlamydomonas reinhardtii* minus mating type is controlled by two genes, *MID* and *MTD1*. Genetics.

[19] Roy SW (2021). Digest: Three sexes from two loci in one genome: a haploid alga expands the diversity of trioecious species. Evolution.

[20] Vurture GW (2017). GenomeScope: fast reference-free genome profiling from short reads. Bioinformatics.

[21] Ferris PJ, Armbrust EV, Goodenough UW (2002). Genetic structure of the mating-type locus of *Chlamydomonas reinhardtii*. Genetics.

[22] Kawai-Toyooka H (2014). Sex-specific posttranslational regulation of the gamete fusogen *GCS1* in the isogamous volvocine alga *Gonium pectorale*. Eukaryot. Cell.

[23] Scott JG (2014). Genome of the house fly, *Musca domestica* L., a global vector of diseases with adaptations to a septic environment. Genome Biol..

[24] Hamm RL, Meisel RP, Scott JG (2015). The evolving puzzle of autosomal versus Y-linked male determination in *Musca domestica*. G3 (Bethesda, Md.).

[25] Meisel RP (2016). Is multifactorial sex determination in the house fly, *Musca domestica* (L.), stable over time?. J. Heredity.

[26] Prochnik SE (2010). Genomic analysis of organismal complexity in the multicellular green alga *Volvox carteri*. Science.

[27] Korneev SA, Park J, O’Shea M (1999). Neuronal expression of neural nitric oxide synthase (nNOS) protein is suppressed by an antisense RNA transcribed from an NOS pseudogene. J. Neurosci..

[28] Tam OH (2008). Pseudogene-derived small interfering RNAs regulate gene expression in mouse oocytes. Nature.

[29] Hanschen ER, Herron MD, Wiens JJ, Nozaki H, Michod RE (2018). Repeated evolution and reversibility of self-fertilization in the volvocine green algae. Evolution.

[30] Lindsey CR, Rosenzweig F, Herron MD (2021). Phylotranscriptomics points to multiple independent origins of multicellularity and cellular differentiation in the volvocine algae. BMC Biol..

[31] Kawachi, M. et al. MCC-NIES List of Strains, 9th Edition. Microbial Culture Collection at National Institute for Environmental Studies, Tsukuba, Japan. 2013.

[32] Starr RC, Zeikus JA (1993). UTEX—The culture collection of algae at the University of Texas at Austin 1993 list of cultures. J. Phycol..

[33] Nozaki H, Ott FD, Coleman AW (2006). Morphology, molecular phylogeny and taxonomy of two new species of *Pleodorina* (Volvoceae, Chlorophyceae). J. Phycol..

[34] Kato S (1982). Laboratory culture and morphology of *Colacium vesiculosum* Ehrb. (Euglenophyceae). Jpn. J. Phycol..

[35] Nozaki H, Kuroiwa H, Mita T, Kuroiwa T (1989). *Pleodorina japonica* sp. nov. (Volvocales, Chlorophyta) with bacteria-like endosymbionts. Phycologia.

[36] Vaster R, Šikić M (2021). Time- and memory-efficient genome assembly with Raven. Nat. Comput. Sci..

[37] Chen S, Zhou Y, Chen Y, Gu J (2018). fastp: an ultra-fast all-in-one FASTQ preprocessor. Bioinformatics.

[38] Hu J, Fan J, Sun Z, Liu S (2020). NextPolish: a fast and efficient genome polishing tool for long-read assembly. Bioinformatics.

[39] Kolmogorov M, Yuan J, Lin Y, Pevzner PA (2019). Assembly of long, error-prone reads using repeat graphs. Nat. Biotechnol..

[40] Hu, J. et al. An efficient error correction and accurate assembly tool for noisy long reads. Preprint at bioRxiv 10.1101/2023.03.09.531669 (2023). 10.1186/s13059-024-03252-4 PMC11046930 38671502

[41] Chakraborty M, Baldwin-Brown JG, Long AD, Emerson JJ (2016). Contiguous and accurate de novo assembly of metazoan genomes with modest long read coverage. Nucleic Acids Res..

[42] Xu M (2020). TGS-GapCloser: a fast and accurate gap closer for large genomes with low coverage of error-prone long reads. Gigascience.

[43] Walker BJ (2014). Pilon: an integrated tool for comprehensive microbial variant detection and genome assembly improvement. PLoS One.

[44] Roach MJ, Schmidt SA, Borneman AR (2018). Purge Haplotigs: Allelic contig reassignment for third-gen diploid genome assemblies. BMC Bioinforma..

[45] Parks DH, Imelfort M, Skennerton CT, Hugenholtz P, Tyson GW (2015). CheckM: assessing the quality of microbial genomes recovered from isolates, single cells, and metagenomes. Genome Res..

[46] Flynn JM (2020). RepeatModeler2 for automated genomic discovery of transposable element families. Proc. Natl Acad. Sci. USA.

[47] Manni M, Berkeley MR, Seppey M, Simão FA, Zdobnov EM (2021). BUSCO update: novel and streamlined workflows along with broader and deeper phylogenetic coverage for scoring of eukaryotic, prokaryotic, and viral genomes. Mol. Biol. Evol..

[48] Palmer J, Stajich J (2019). nextgenusfs/funannotate: funannotate v1.5.3 (1.5.3). Zenodo.

[49] Camacho C (2009). BLAST+: architecture and applications. BMC Bioinforma..

[50] Noe L, Kucherov G (2005). YASS: enhancing the sensitivity of DNA similarity search. Nucleic Acids Res..

[51] Edgar RC (2004). MUSCLE: multiple sequence alignment with high accuracy and high throughput. Nucleic Acids Res..

[52] Felesenstein J (1985). Confidence limits on phylogenies: an approach using the bootstrap. Evolution.

[53] Kumar S, Stecher G, Li M, Knyaz C, Tamura K (2018). MEGA X: molecular evolutionary genetics analysis across computing platforms. Mol. Biol. Evol..

[54] Ronquist F (2012). MRBAYES 3.2: efficient Bayesian phylogenetic inference and model selection across a large model space. Syst. Biol..

[55] Darriba D (2020). ModelTest-NG: a new and scalable tool for the selection of DNA and protein evolutionary models. Mol. Biol. Evol..

[56] Vos RA (2012). NeXML: rich, extensible, and verifiable representation of comparative data and metadata. Syst. Biol..

[57] Yang Z (2007). PAML 4: phylogenetic analysis by maximum likelihood. Mol. Biol. Evol..

[58] Yang Z, Nielsen R (2000). Estimating synonymous and nonsynonymous substitution rates under realistic evolutionary models. Mol. Biol. Evol..

[59] Taylor SC (2019). The ultimate qPCR experiment: producing publication quality, reproducible data the first time. Trends Biotechnol..

[60] Okido T (2021). DNA Data Bank of Japan (DDBJ) update report 2021. Nucleic Acids Res..

