# Supplementary material for: Reorganization of the ancestral sex-determining regions during the evolution of trioecy in Pleodorina starrii
Source: Commun Biol. 2023 Jun 9;6:590. doi: 10.1038/s42003-023-04949-1 (PMC10256686; doi:10.1038/s42003-023-04949-1)
Supplement: Supplementary file 2 — Supplementary Information [file 42003_2023_4949_MOESM2_ESM.pdf]

Supplementary Information for

Reorganization of the ancestral sex-determining regions during the evolution of trioecy in *Pleodoorina starrii*

Kohei Takahashi,[1] Shigekatsu Suzuki,[2] Hiroko Kawai-Toyooka,[3] Kayoko Yamamoto,[4] Takashi Hamaji,[5] Ryo Ootsuki,[4,6] Haruyo Yamaguchi,[2] Masanobu Kawachi,[2] Tetsuya Higashiyama,[1] and Hisayoshi Nozaki[1,2*]

[1] Department of Biological Sciences, Graduate School of Science, The University of Tokyo, Hongo, Bunkyo-ku, Tokyo 113-0033, Japan
[2] Biodiversity Division, National Institute for Environmental Studies, Onogawa, Tsukuba, Ibaraki 305-8506, Japan.
[3] Department of Frontier Bioscience, Hosei University, Kajino-cho, Koganei, Tokyo 184-8584, Japan
[4] Department of Chemical and Biological Sciences, Faculty of Science, Japan Women's University, Bunkyo-ku, Tokyo 112-8681, Japan
[5] Research and Development Initiative, Chuo University, Kasuga, Bunkyo-ku, Tokyo 112-8551, Japan
[6] Department of Natural Sciences, Faculty of Arts and Sciences, Komazawa University, Komazawa, Setagaya-ku, Tokyo 154-8525, Japan

*Corresponding Author: Hisayoshi Nozaki
Email: Hisayoshi.nozaki@gmail.com

Supplementary Information

This file contains Supplementary Figures 1-16, Supplementary Tables 1-10, and Supplementary References.

Table of Contents

Supplementary Figures 1-16 --- 3
Supplementary Tables 1-10 --- 19
Supplementary References -- 28

Supplementary Figures

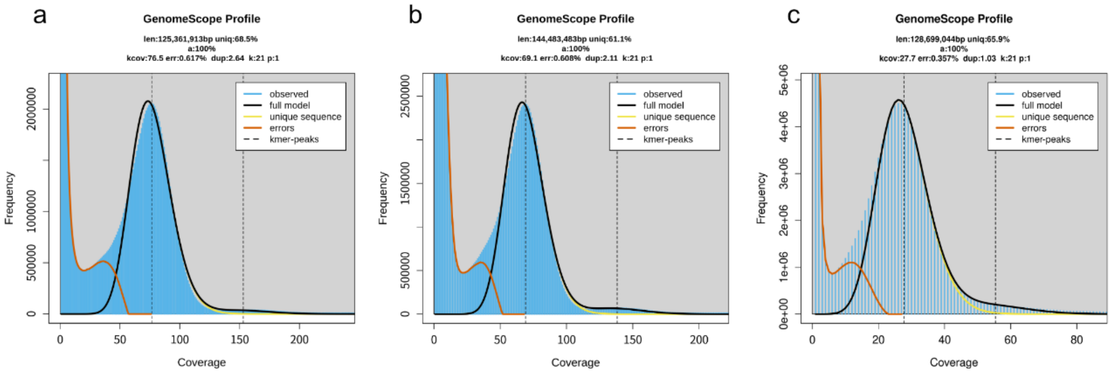

Supplementary Figure 1 | GenomeScope profiles[1] showing *k-mer* frequencies in *Pleodorina starrii*. For details of the frequencies, see Supplementary Table 3. (a) Unisexual male phenotype (NIES-1363). (b) Unisexual female phenotype (NIES-4481). (c) Bisexual phenotype (NIES-4479).

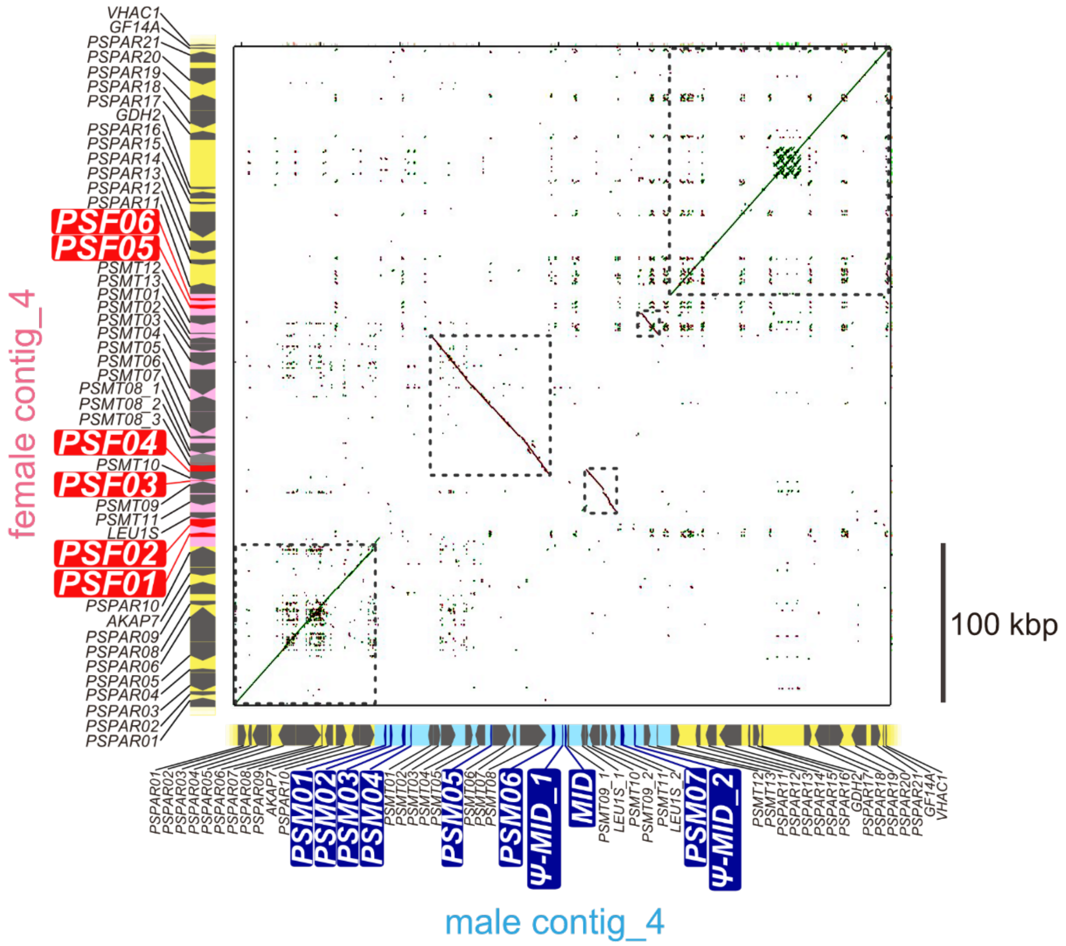

Supplementary Figure 2 | Dotplot between male contig_4 (horizontal) and female contig_4 (vertical) around sex-determining regions [male SDR (light blue) and female SDR (pink), respectively] and part of pseudo autosomal regions (yellow) of *Pleodorina starrii*. Green and red dots indicate forward and reverse alignments, respectively.

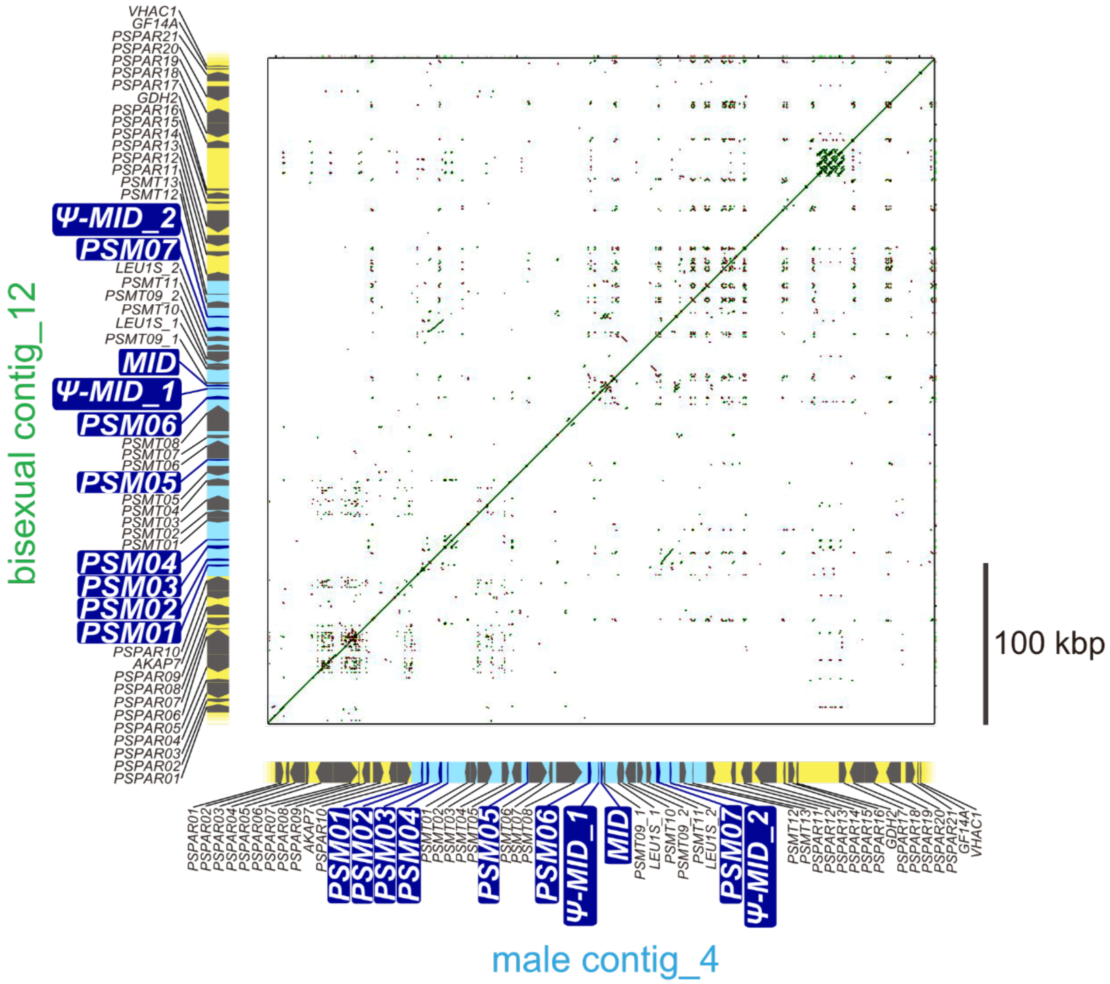

Supplementary Figure 3 | Dotplot between male contig_4 (horizontal) and bisexual contig_12 (vertical) around sex-determining regions [male SDRs (light blue)] and part of pseudo autosomal regions (yellow) in *Pleodorina starrii.* Green and red dots indicate forward and reverse alignments, respectively.

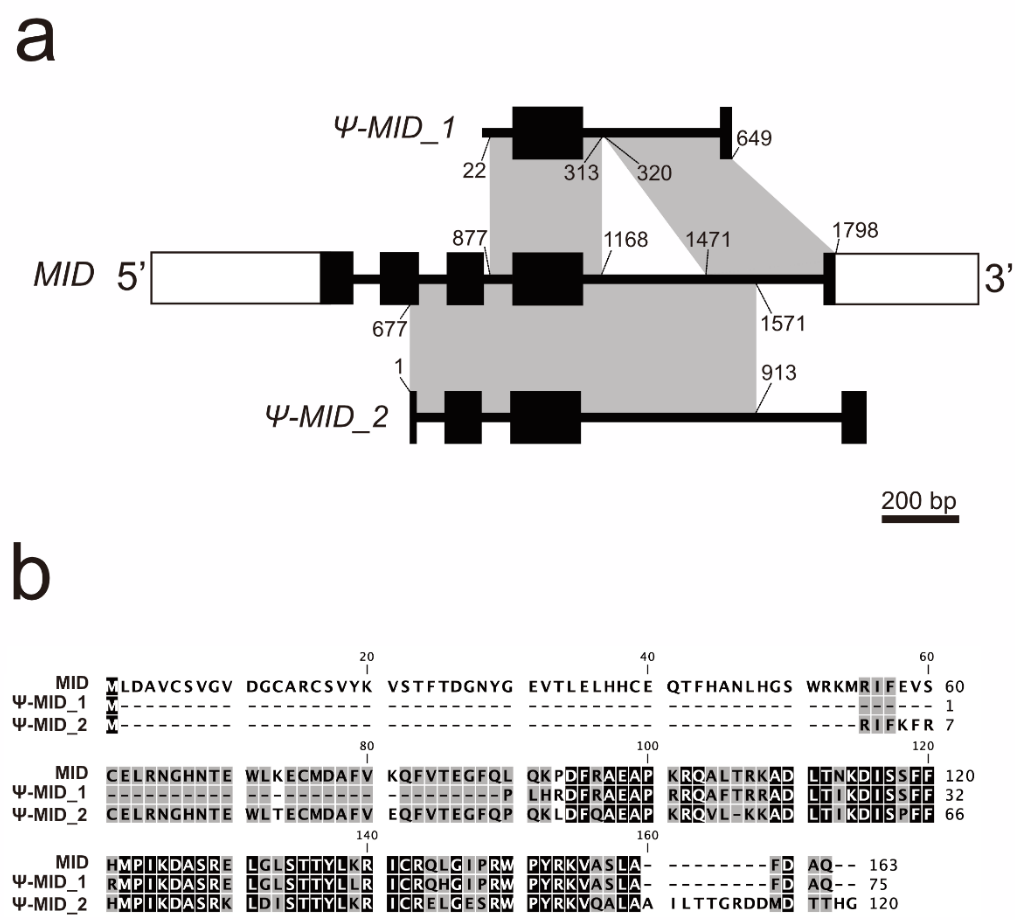

Supplementary Figure 4 | Comparison of three paralogs of *MID* in male SDR of *Pleodorina starrii*. (a) Exon-intron structures of the *MID*[2] (AB272616), *ψ -MID_1*, ψ-*MID_2* identified in this study. Note that filled and open boxes represent coding and non-coding exon sequences, respectively. Numbers above and below boxes indicate corresponding nucleotide numbers. Gray boxes link homologous sequences. (b) Alignment of deduced amino acid sequences of three *MID* paralogs in *P. starrii* using CLC Sequence Viewer 8.0 (QIAGEN Aarhus A/S). Residues identical in all three sequences are shaded in black, and residues identical in two sequences are shaded in gray.

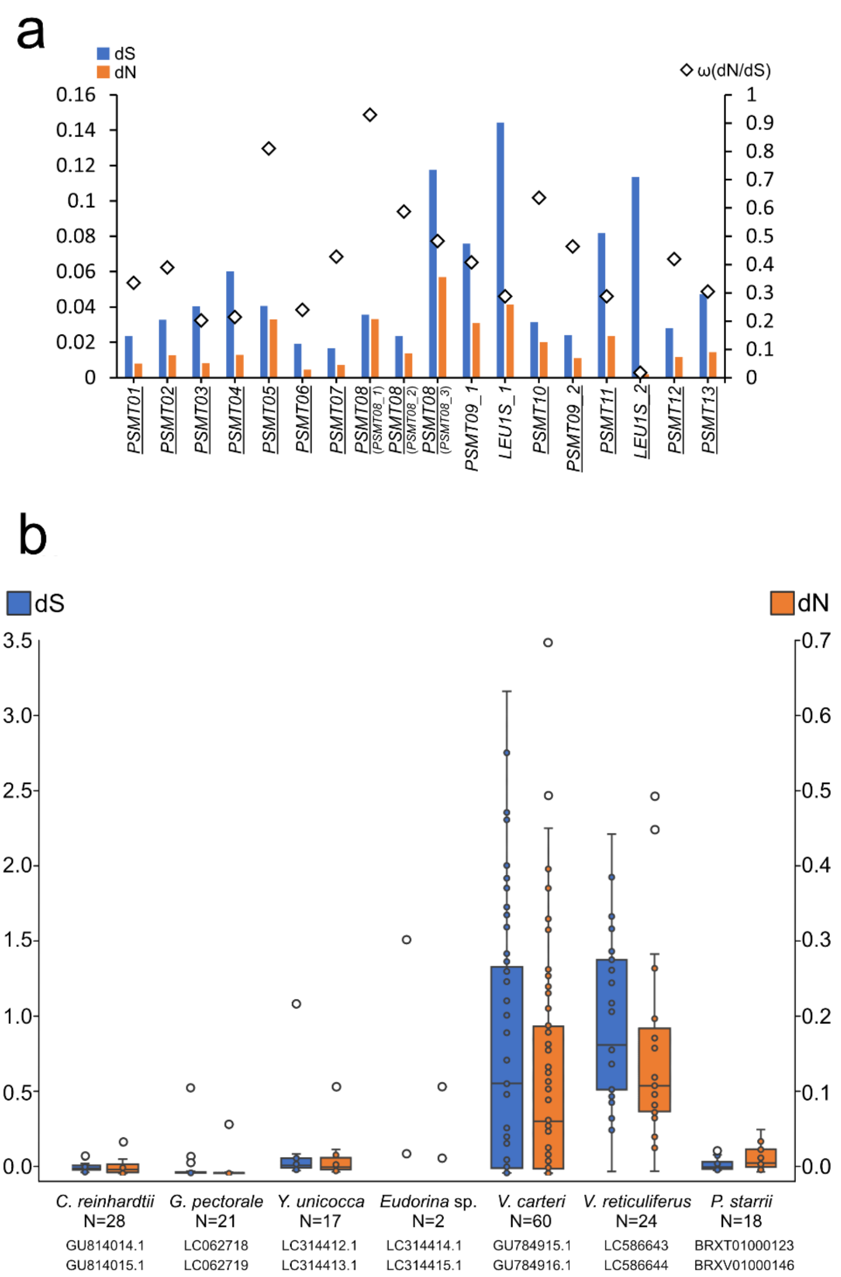

Supplementary Figure 5 | Molecular evolutionary analyses of gametologs in *Pleodorina starrii*. (a) Non-synonymous and synonymous ratios (dN and dS, respectively) of gametologs between male and female sex-determining regions (SDRs) of *P. starrii*. Note that the majority of the gametologs (underlined) localized within the inverted regions (Fig. 2a; Supplementary Fig. 3). There are no gametologs showing dN/dS > 1 between sexes. For codon-based analysis of positive selection of *LEU1S,* see Supplementary Table 4. (b) Box-whisker plots comparing the distributions of dS (blue/left) and dN (orange/right) for gametolog pairs found in SDRs of volvocine algal haploid sex chromosomes. Dots are outliers from interquartile ranges. For phylogeny of the typical gametolog *LEU1*, see Supplementary Fig. 6.

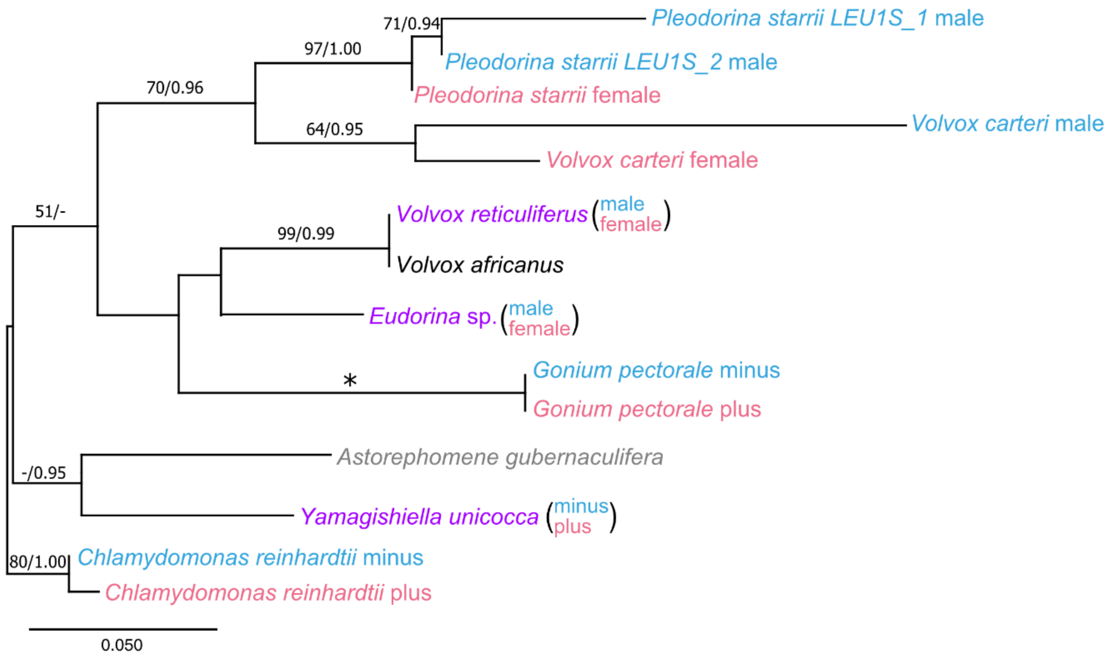

Supplementary Figure 6 | Phylogeny of homologs of *LEU1S* in the volvocine lineage, inferred based on 132 deduced amino acid sequences by maximum likelihood (ML) method using the model [WAG[3]+ G model] selected by MEGAX[4]. Blue and pink represent gametologs from mating type minus/male and mating type plus/female SDRs, respectively. Purple indicates autosomal genes harbored in all sex phenotypes of the species. Black represents homothallic species. Gray shows species with unknown sex phenotypes. All positions containing gaps and missing data were eliminated from the alignment. Branch lengths are proportional to the evolutionary distances indicated by the scale bar. Numbers at left and right above branches indicate bootstrap values (BV) of the ML (≧50%) and posterior probabilities (PP) of Bayesian inference (≧0.90), respectively. Asterisks at the branches indicate 100% BV and 1.00 PP by the two methods.

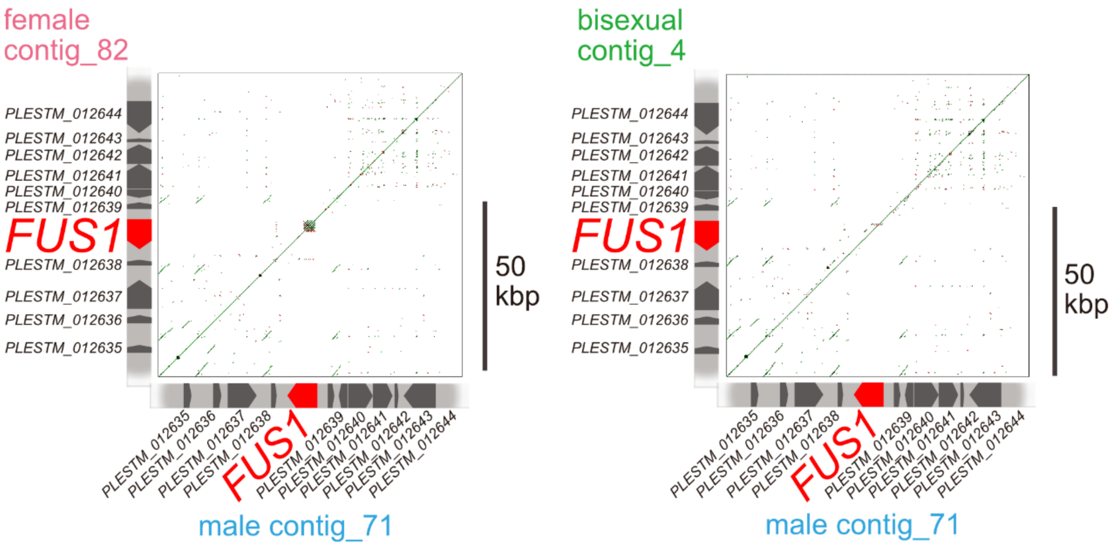

Supplementary Figure 7 | Dotplots of autosomal regions harboring *FUS1* and adjacent genes between between male (horizontal) and female/bisexual (vertical) phenotypes. Green and red dots indicate forward and reverse alignments, respectively.

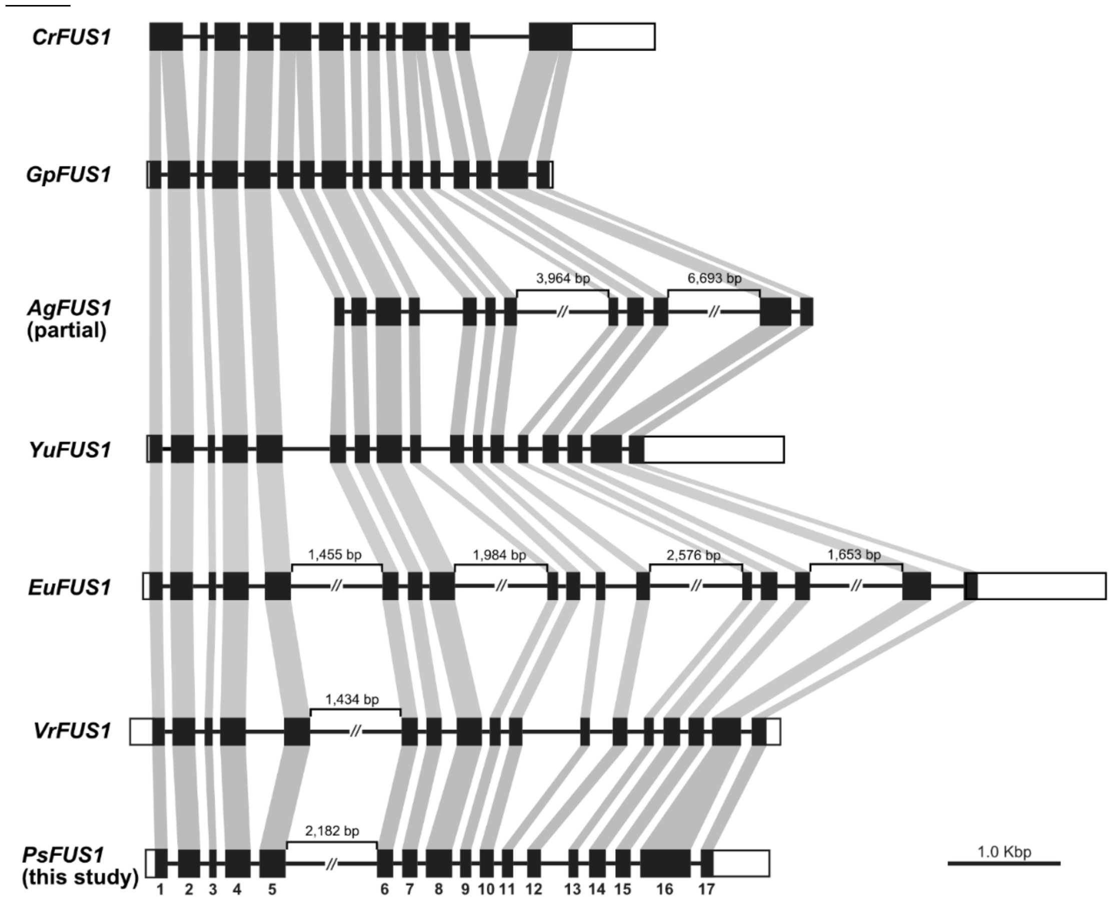

Supplementary Figure 8 | Exon-intron structures of *FUS1* orthologs from *Chlamydomonas reinhardtii*[5](*CrFUS1*), *Gonium pectorale*[6](*GpFUS1*), *Astrephomene gubernaculifera*[7](*AgFUS1*), *Yamagishiella unicocca*[8](*YuFUS1*), *Eudorina* sp.[8] (*EuFUS*), *Volvox reticuliferus*[9] (*VrFUS1*), and *Pleodorina starrii* (*PsFUS1*) are shown for comparison. For details *PsFUS1*, see Fig. 2. Filled and open boxes represent coding and non-coding exon sequences, respectively. Numbers below boxes for *PsFUS1* indicate exon numbers. Gray boxes link homologous coding sequences. The accession numbers of sequences used in this figure are summarized in Supplementary Table 8.

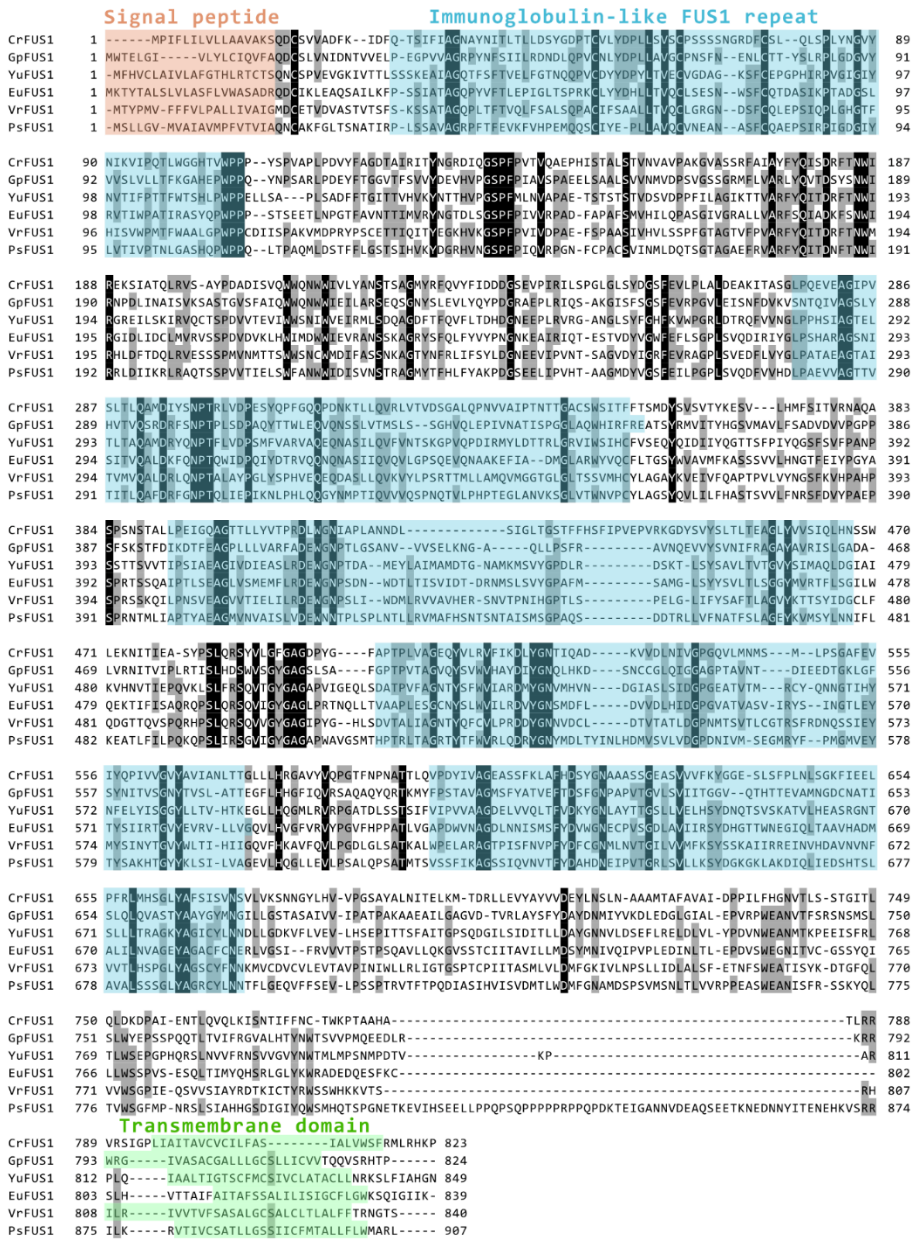

Supplementary Figure 9 | Alignment of the deduced *FUS1* amino acid sequences. The *FUS1* sequences of *Chlamydomonas reinhardtii* (*CrFUS1*), *Gonium pectorale* (*GpFUS1*), *Yamagishiella unicocca* (*YuFUS1*), *Eudorina* sp. (*EuFUS1*), *Volvox reticuliferus* (*VrFUS1*), and *Pleodorina starrii* (*PsFUS1*, identified in this study) were aligned using the MUSCLE program[10] built in the GENETYX-MAC software ver. 20.1.1 (Genetyx Co.) with manual adjustments. Residues identical in all six sequences are shaded in black, and residues identical in four or five sequences are shaded in gray. The immunoglobulin-like *FUS1* repeats are shaded in blue. The putative signal peptide and transmembrane domain in each sequence were predicted by Phobius 3[11] and shaded in reddish brown and green, respectively. The accession numbers of sequences used in this figure are summarized in Supplementary Table 8.

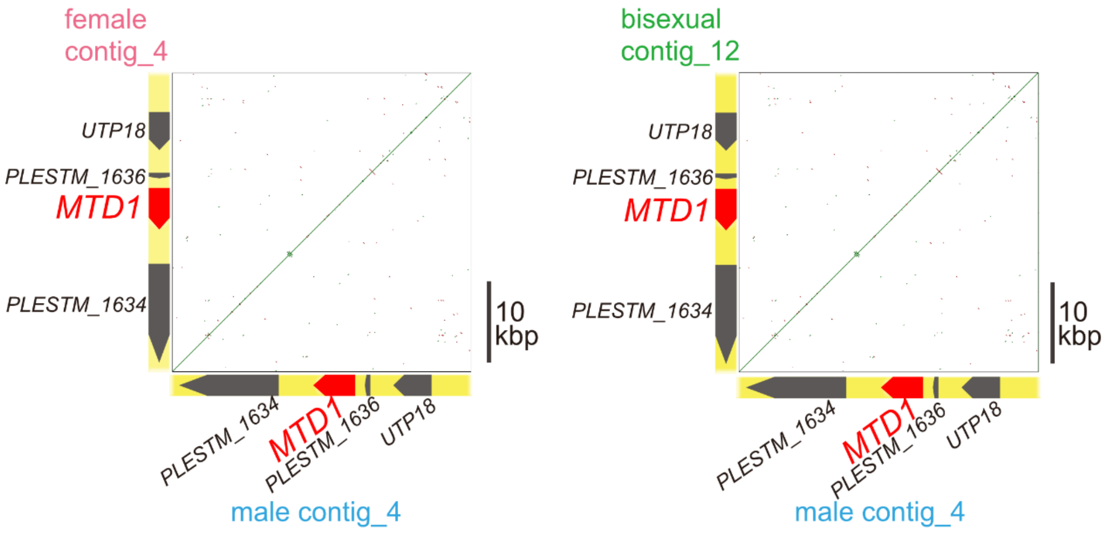

Supplementary Figure 10 | Dotplots between pseudo autosomal regions harboring *MTD1* and adjacent genes of three sex phenotypes of *Pleodorina starrii.* Green and red dots indicate forward and reverse alignments, respectively.

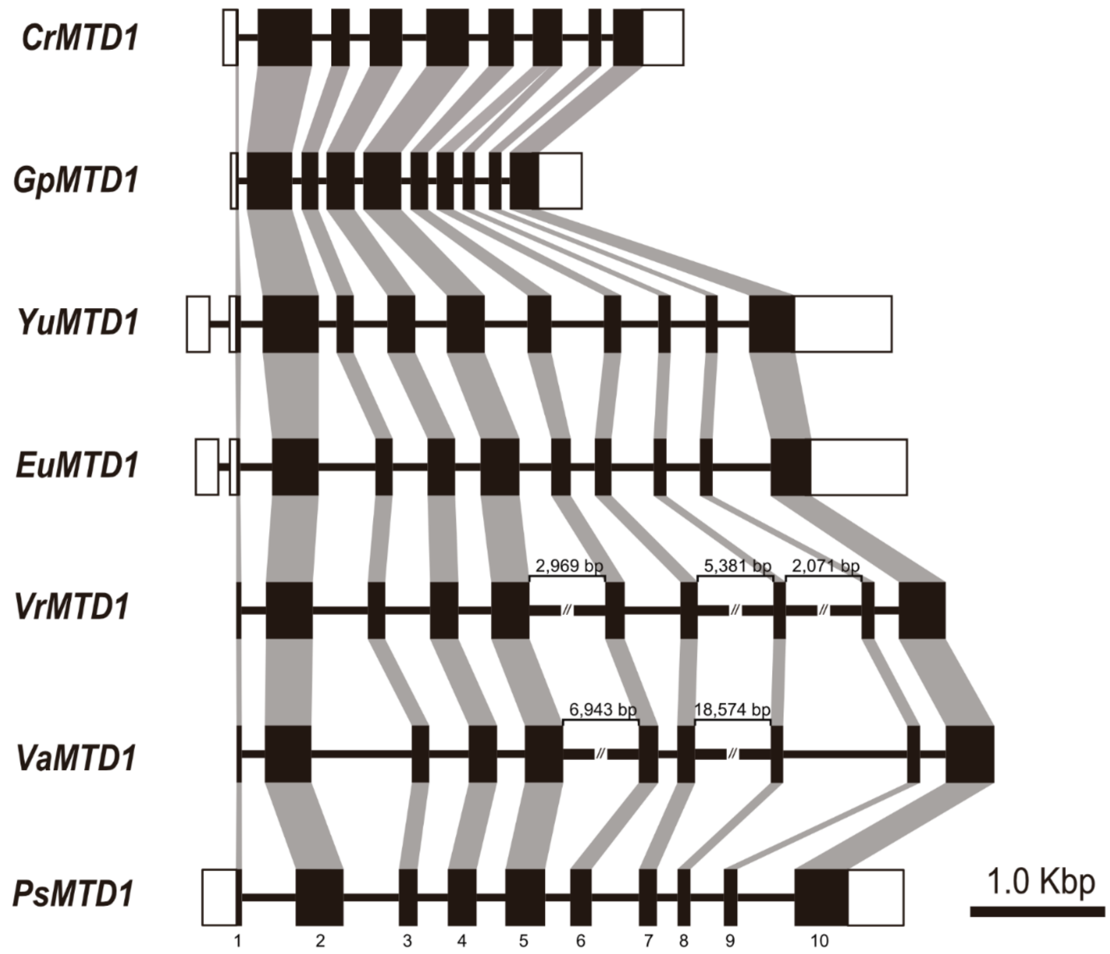

Supplementary Figure 11 | Exon-intron structures of the *MTD1* orthologs from *Chlamydomonas reinhardtii*[12](*CrMTD1*), *Gonium pectorale*[13](*GpMTD1*), *Yamagishiella unicocca*[8](*YuMTD1*), *Eudorina* sp.[8] (*EuFUS1*), *Volvox reticuliferus*[9] (*VrMTD1*), *Volvox africanus*[9] (*VaMTD1*), and *Pleodorina starrii* (*PsMTD1*) are shown for comparison. For details of *PsMTD1*, see Fig. 2. Filled and open boxes represent coding and non-coding exon sequences, respectively. Numbers below boxes for *PsMTD1* indicate exon numbers. Gray boxes link homologous coding sequences. The accession numbers of sequences used in this figure are summarized in Supplementary Table 8.

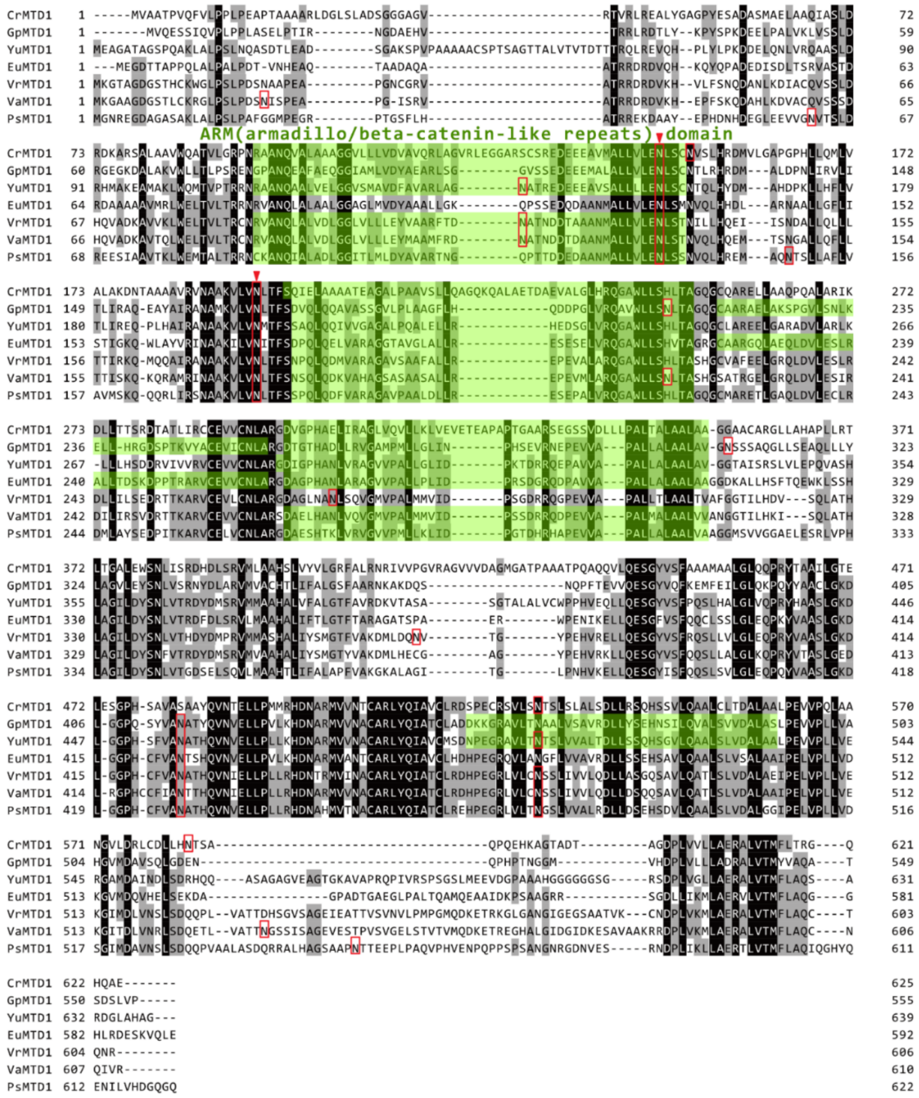

Supplementary Figure 12 | Alignment of the deduced *MTD1* amino acid sequences from seven volvocine species. The *MTD1* sequences of *Chlamydomonas reinhardtii* (*CrMTD1*), *Gonium pectorale* (*GpMTD1*), *Yamagishiella unicocca* (*YuMTD1*), *Eudorina* sp. (*EuMTD1*), *Volvox reticuliferus* (*VrMTD1*), *Volvox africanus* (*VaMTD1*) and *Pleodorina starrii* (*PsMTD1*, identified in this study) were aligned using the MUSCLE program built in the GENETYX-MAC software ver. 20.1.1 (Genetyx Co.) with manual adjustments. Residues identical in all seven sequences are shaded in black, and residues identical in four, five, or six sequences are shaded in gray. Armadillo/beta-catenin-like repeat (ARM) domains were predicted by the genomic mode of the SMART database[14,15] and shaded in light green. Potential N-glycosylation sites were predicted by GlycoEP[16] and marked with red boxes. Red arrowheads above the boxes indicate the N-glycosylation sites conserved in all seven proteins. The accession numbers of sequences used in this figure are summarized in Supplementary Table S8.

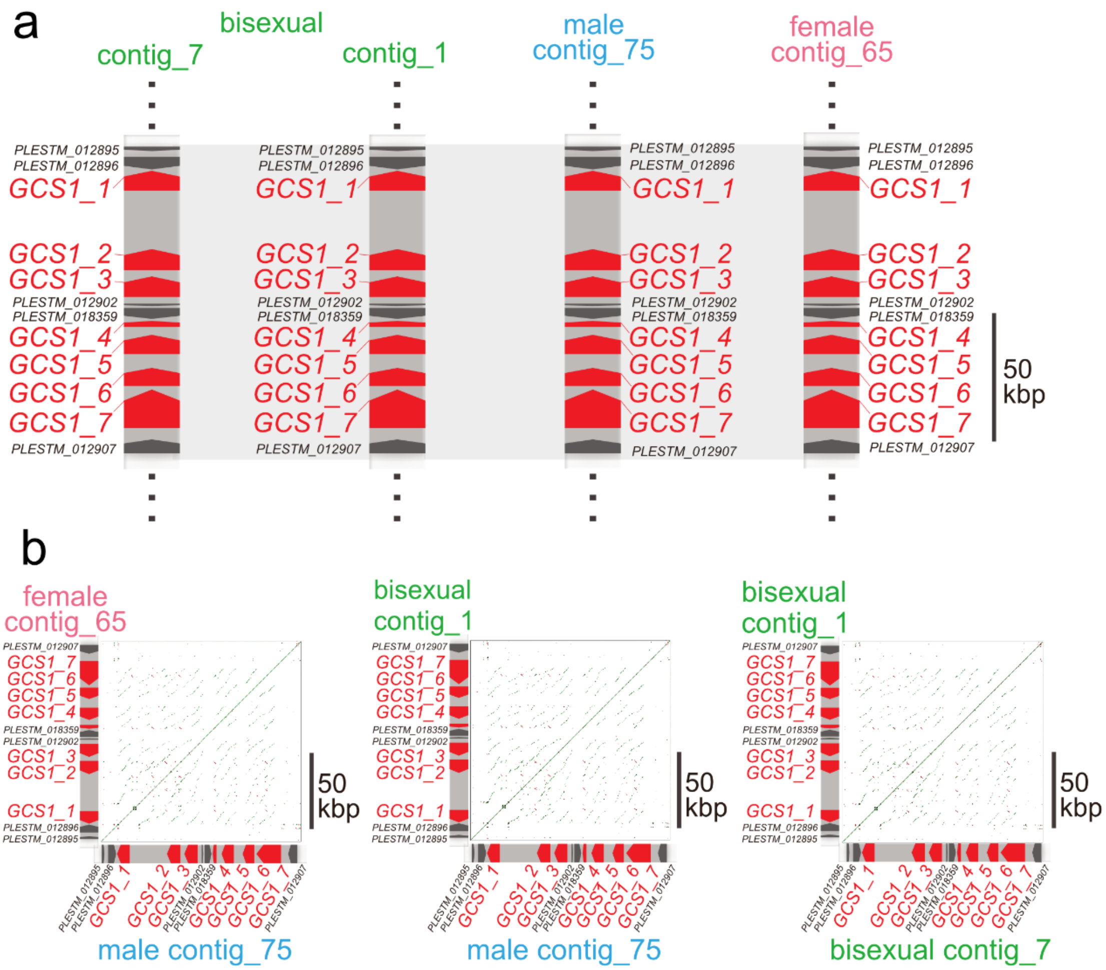

Supplementary Figure 13 | Autosomal regions harboring *GCS1* and adjacent genes of *Pleodorina starrii.* (a) Comparison between male (contig_75), female (contig_65), and bisexual (contig_1, contig_7) sex phenotypes. Gray shading indicates a syntenic bloc. (b) Dotplots between male (horizontal) and female/bisexual (vertical) phenotypes. Green and red dots indicate forward and reverse alignments, respectively.

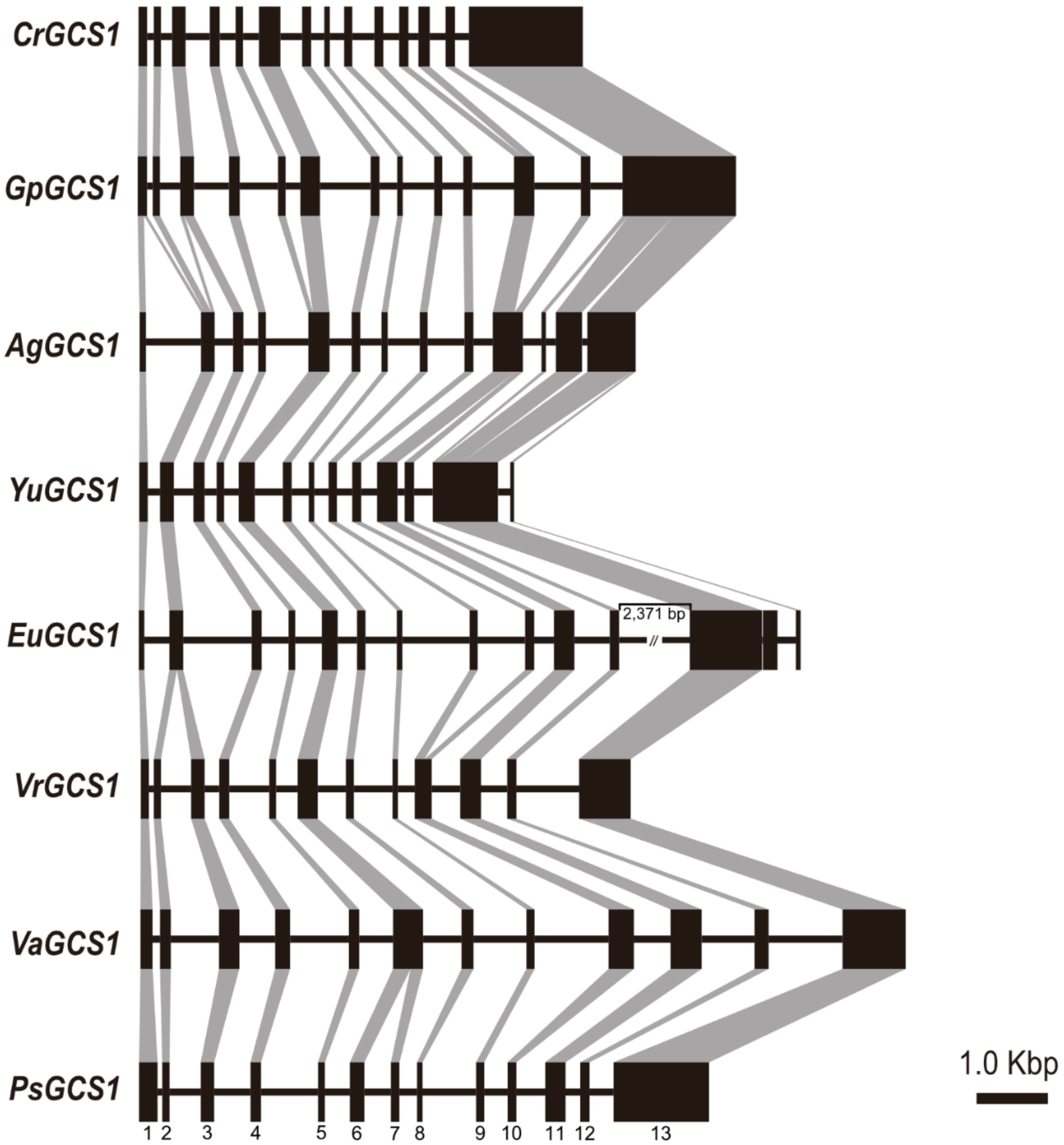

Supplementary Figure 14 | Exon-intron structures of the *GCS1* orthologs from *Chlamydomonas reinhardtii*[17] (*CrGCS1*), *Gonium pectorale*[18] (*GpGCS1*), *Astrephomene gubernaculifera*[7] (*AgGCS1*), *Yamagishiella unicocca*[8] (*YuGCS1*), *Eudorina* sp.[8] (*EuGCS1*), *Volvox carteri*[19] (*VcGCS1*), *Volvox reticuliferus*[9] (*VrGCS1*), *Volvox africanus*[9] (*VaGCS1*), and *Pleodorina starrii* (*PsGCS1=GCS1_1*) are shown for comparison. For details of *PsGCS1*, see Fig. 3. Filled boxes represent coding exon sequences. Numbers below boxes for *PsGCS1* indicate exon numbers. Gray boxes link homologous coding sequences. The accession numbers of sequences used in this figure are summarized in Supplementary Table 8.

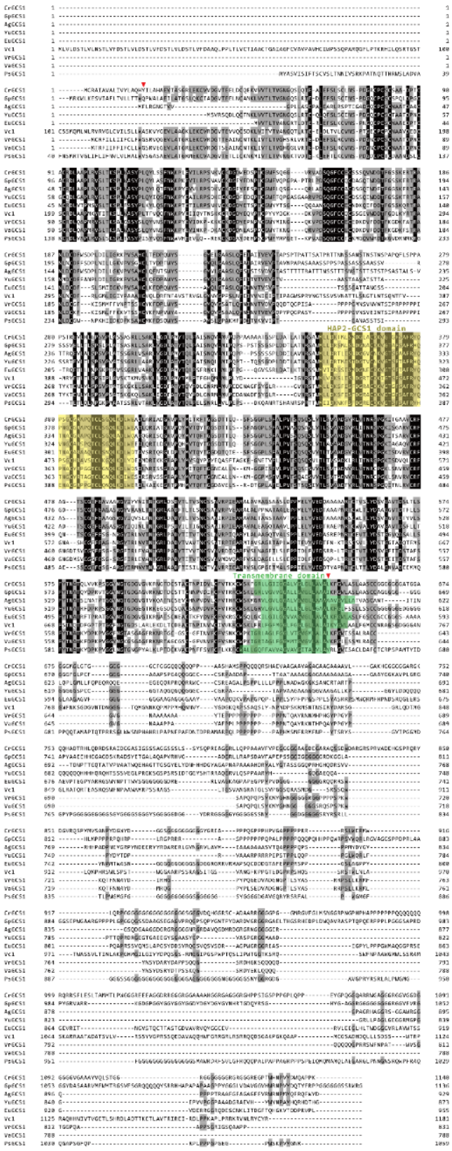

Supplementary Figure 15 | Alignment of the deduced *GCS1* amino acid sequences from seven volvocine species. For the seven species, see Supplementary Table 8. The *GCS1* sequences of *Chlamydomonas reinhardtii* (*CrGCS1*), *Gonium pectorale* (*GpGCS1*), *Astorephomene gubernaculifera* (*AgGCS1*), *Yamagishiella unicocca* (*YuGCS1*), *Eudorina* sp. (*EuGCS1*), *Volvox carteri* (*Vc1*), *Volvox reticuliferus* (*VrGCS1*), *Volvox africanus* (*VaGCS1*, accession number GIL53993) and *Pleodorina starrii* (*PsGCS1=GCS1_1*, Fig. 3) were aligned using the MUSCLE program built in the GENETYX-MAC software ver. 20.1.1 (Genetyx Co.) with manual adjustments. Residues identical in all 9 sequences are shaded in black, and residues identical in 5, 6, 7, or 8 sequences are shaded in gray. The HAP2-GCS1 domains were predicted by the InterPro 90.0[20] and shaded in yellow. Potential N-glycosylation sites were predicted by GlycoEP[16] and marked with red boxes. Red arrowheads above the boxes indicate the N-glycosylation sites conserved in all seven proteins. The putative transmembrane domain in each sequence were predicted by Phobius 3[11] and shaded in green. Red arrowheads indicate the start and end positions of the sequences used to create the *GCS1* molecular phylogenetic tree (Fig. 3c).

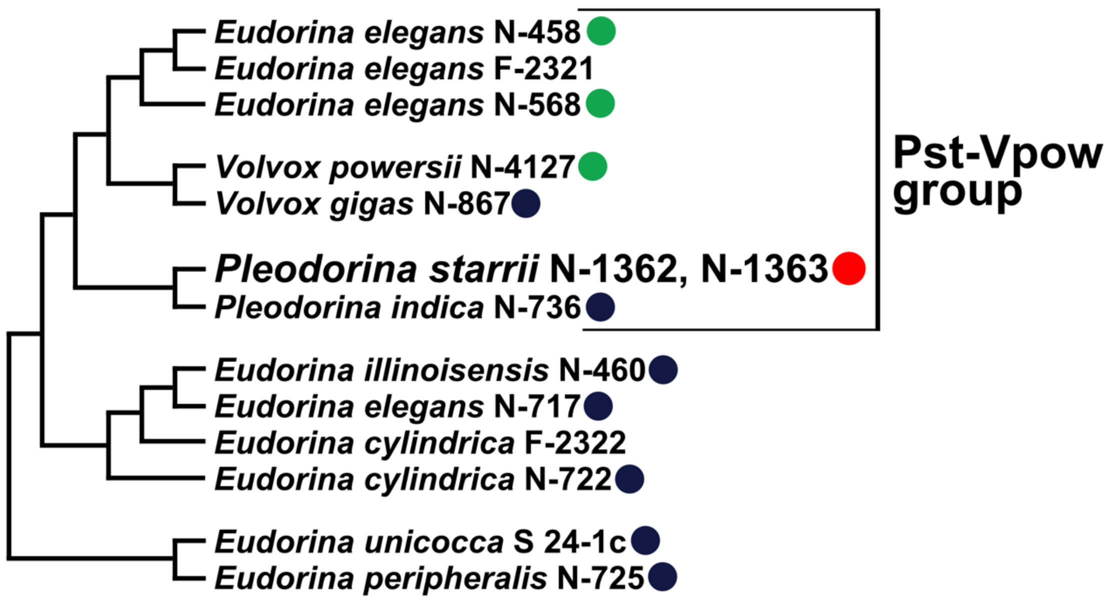

Supplementary Figure 16 | Diagram of phylogenetic position of *Pleodorina starrii* within part of the anisogamous/oogamous volvocine algae. The phylogeny is based on Lindsey *et al.*[21]. Navy and green circles represent heterothallic and homothallic species/strains, respectively. Red circle represents trioecious species. “N” represents strains of Microbial Culture Collection at the National Institute for Environmental Studies (NIES Collection, Japan). “S” represents strains of Culture Collection of Algae at Göttingen University (SAG, Germany). “F” represents strains of The Freshwater Algae \culture Collection at the Institute of Hydrobiology, Chinese Academy of Sciences (FACHB, China).

Supplementary Tables

Supplementary Table 1. Five types of mating systems in diploid species.

Diploid mating System	Dioecy	Monoecy (hermaphrodite, cosexuality)	Androdioecy	Gynodioecy	Trioecy
Sex phenotype	unisexual male and unisexual female individuals	bisexual individual	unsexual male and bisexual individuals	unisexual female and bisexual individuals	unisexual male, unisexual female, and bisexual individuals
Example (flowering plant and/or metazoan)	*Silene latifolia*, most vertebrates	most flowering plants, many invertebrates	*Caenorhabditis elegans*, *Goniastrea australensis*	*Silene vulgaris*, *Lobelia siphilitica*	*Carica papaya*, *Auanema rhodensis*
References	Renner[22], Bachtrog *et al.*[23]		Kojis & Quinn[24], Ward & Carrel[25]	Joll & Chenier[26], Molano-Flores[27]	Wang *et al.*[28], Kanzaki *et al.*[29]

Supplementary Table 2. Three types of mating systems in haploid species.

Haploid mating system	Heterothallism	Homothallism	Trioecy
Sex phenotype and genotype of clonal culture	unisexual male and unisexual female by different genotypes	bisexual by a single genotype	unisexual male, unisexual female, and bisexual by different genotypes
References	Bold *et al.*[30]		Takahashi *et al.*[31], Roy[32]

Supplementary Table 3. Comparison of details of whole nuclear genomes and sex-determining regions (SDRs) between three sexual phenotypes of *Pleodorina starrii*.

Strain designation	NIES-1363		NIES-4481		NIES-4479	
Sex	Male		Female		Bisexual	
	Whole genome	SDR	Whole genome	SDR	Whole genome	SDR
Total length [bp]	134,838,970	184,776	134,979,981	136,796	136,124,605	184,778
Number of contigs	242	—	288	—	95	—
Min [bp]	17,603	—	14,430	—	44,988	—
Max [bp]	3,832,317	—	4,523,807	—	10,277,549	—
Average [bp]	557,186	—	468,680	—	1,432,891	—
Contig N50 [bp]	1,064,554	—	833,246	—	3,124,857	—
GC [%]	62.89	60.53	62.82	62.42	62.89	60.54
Number of genes* (sex-specific genes, gametologs)	18,399	24 (8,16)	17,356	22 (6,16)	18,572	24 (8,16)
Gene density [genes/Mbp]	136.5	129.9	128.6	160.8	136.4	129.9
Repeats [%]**	29.34	33.24	29.77	28.62	27.77	33.77
BUSCO scores***	C: 98.2% [S: 97.5%, D: 0.7%], F: 0.8%, M:1.0%	—	C: 97.3% [S: 96.4%, D: 0.9%], F: 0.7%, M:2.0%	—	C: 97.5% [S: 96.8%, D: 0.7%], F: 0.7%, M:1.8%	—

*Excluding pseudogene.

**Repetitive sequences identified using RepeatMasker v4.1.2-p1 and RepeatModeler v2.0.3[33] with -xsmall option.

***BUSCO v5.3.2[34] scores calculated based on chlorophyta_obd10 (1,519 BUSCO). C: complete; S: complete and single-copy; D: complete and duplicated; F: fragmented; M: missing.

Supplementary Table 4. Codon-based analysis of positive selection in *LEU1S* genes (Supplementary Fig. 6).

Model	Positively selected sites*
M2a (positive selection)[35,36]	not detected
M8 (beta & ω)[37]	not detected

*Based on the codon-based analysis of 132 codons of 14 *LEU1S* homologs of the volvocine algae (Supplementary Fig. 6) by codeml of the PAML4 package[38] (version 4.9j).

Supplementary Table 5. Comparison of whole genome and SDRs properties of the volvocine algae.

whole genome						
Species	Mating type /sex	Size/ total length [Mbp]	GC [%]	Number of genes	Gene density [genes/ Mbp]	DDBJ accessions no.
Pleodorina starrii	Male	134.8	62.9	18,154	134.6	BRXT01000001 -BRXT01000242
	Female	135.0	62.8	17,110	126.8	BRXV01000001 -BRXV01000288
	Bisexual	136.1	62.9	18,293	134.4	BRXU01000001 -BRXU01000095
Volvox reticuliferus	Male	134.0	54	14,050	104.8	BNCQ01000001 -BNCQ1000230
	Female	133.0	54	13,860	104.2	BNCP01000001 -BNCP01000200
V. africanus	Bisexual	129.3	53	13,716	108.1	BNCO01000001 -BNCO01000448
V. carteri	Male	N.d.*	N.d.	N.d.	N.d.	N.d.
	Female	131.1	56.1	14,958	114.1	GCA_000143455.1
Eudorina sp.	Male	168.6	61.3	N.d.	N.d.	GCA_003117095.1
	Female	184.0	61.0	N.d.	N.d.	GCA_003117195.1
Yamagishiella unicocca	Minus	140.8	60.8	N.d.	N.d.	GCA_003117035.1
	Plus	134.2	61.1	N.d.	N.d.	GCA_003116995.1
Gonium pectorale	Minus	N.d.	N.d.	N.d.	N.d.	N.d.
	Plus	148.8	64.5	17990	120.9	GCA_001584585.1
Chlamydomo-nas reinhardtii	Minus	N.d.	N.d.	N.d.	N.d.	N.d.
	Plus	111.1	64.1	17741	159.7	GCA_000002595.2

SDR or SDLR/short SDLR						
Species	Mating type /sex	Size [Mbp]	GC [%]	Number of genes (**)	Gene density [genes/ Mbp]	DDBJ accessions no.
Pleodorina starrii	Male	0.185	60.5	24(16)	129.9	BRXT01000123
	Female	0.137	62.4	22(16)	160.8	BRXV01000146
	Bisexual	0.185	60.5	24(16)	129.9	BRXU01000012
Volvox reticuliferus	Male	0.98	51	28(25)	27.7	LC586644
	Female	1.01	51	28(25)	28.6	LC586643

Table S5. Continued.

V. africanus	Bisexual	1.02 /0.20	51 /50	30/4	29.4/20	LC586641 /LC586642
V. carteri	Male	1.13	53	60(50)	54	GU784916.1
	Female	1.51	52	55(50)	39	GU784915.1
Eudorina sp.	Male	0.007	51.4	3(2)	428	LC314415.1
	Female	0.09	53.9	3(2)	33.3	LC314414.1
Yamagishiella unicocca	Minus	0.165	60.3	18(17)	109	LC314413.1
	Plus	0.268	60.1	18(17)	67.2	LC314412.1
Gonium pectorale	Minus	0.499	61	24(21)	46	LC062719
	Plus	0.366	59.7	24(21)	58	LC062718
Chlamydomonas reinhardtii	Minus	0.204	61	25(22)	118	GU814015.1
	Plus	0.31	60	35(22)	109	GU814014.1

References: *P. starrii* (the present study); *C. reinhardtii* and *V. carteri*[39]; *G. pectorale*[5]; *Y. unicocca* and *Eudorina* sp.[8]; *V. reticuliferus* and *V. africanus*[9]

*Not determined.

**The number of gametologs in parentheses.

Supplementary Table 6. Characteristics of the genomic illumina and PacBio raw reads of *Pleodorina starrii*.

Species	Strain	Sex phenotype	Total # of raw reads	Total # of clan PE reads	N50 [bp]	GC [%]	Sequence method
Pleodorina starrii	NIES-1363	unisexual male	53,057,984	50,690,357	150	58	Illumina NovaSeq 6000
			1,101,294	-	19,003	58	PacBio sequel II
	NIES-4481	unisexual female	53,641,190	51,454,638	150	56	Illumina NovaSeq 6000
			9,606,655	-	11,687	58	PacBio sequel II
	NIES-4479	Bisexual	56,808,725	54,493,946	150	60	Illumina NovaSeq 6000
			1,663,200	-	20,018	60	PacBio sequel II

Supplementary Table 7. Characteristics of the RNA-seq raw reads of *Pleodorina starrii*.

Species	Strain	Sex phenotype	Condition	Total # of raw reads	Total # of clan PE reads	N50 [bp]	GC [%]	Sequence method
Pleodorina starrii	NIES-4480	unisexual male	asexual	14,781,533	14,649,207	150	61	Illumina NovaSeq 6000
			sexually induced	13,545,405	13,417,465	150	61	
	NIES-4481	unisexual female	asexual	13,997,246	13,841,311	150	63	
			sexually induced	15,585,854	15,366,326	134	61	
	NIES-4482	Bisexual	asexual	21,346,926	21,062,944	150	63	
			sexually induced	16,925,671	16,617,519	150	62	

Supplementary Table 8. List of accession numbers used in phylogenetic analysis in the present study.

Species	*MID*	*FUS1*	*MTD1*	*GCS1*	*LEU1S*
Chlamydomonas reinhardtii	U92701	U49864	AF417574	XM_001695841	GU814014<br>GU814015
Gonium pectorale	AB353340	BAU61607	BAU61585	AB915401	BAU61575<br>BAU61603
Astorephomene gubernaculifera		GFR43143		BMAR01000020	GFR43182
Yamagishiella unicocca	LC274882	BBC28430	LC314416	BDSK01000011	BDSK01000005
Eudorina sp.	LC274881	BBC28482	BBC28487	BDSJ01000037	BDSJ01000021
Pleodorina starrii	AB272616	LC740508*	LC740509*<br>LC740510*<br>LC740511*	LC740512*,<br>LC740513*,<br>LC740514*,<br>LC740515*,<br>LC740516*,<br>LC740517*,<br>LC740518*	LC740519*,<br>LC740520*,<br>LC740521*
Volvox carteri	ADI46915			XM_002952837<br>XM_002952838	ADI46877<br>ADI46907
Volvox reticuliferus	LC274879	BCL66190	BCL66242	GIM10032	BCL66201
Volvox africanus	LC274875		BNCO01000040	GIL53993	BCL66133
Chlamydomonas globose	AF002710				
Gonium maiaprillis	AB623044				
Gonium multicoccum	AB774225<br>AB774226				
Gonium octonaryium	AB774227				
Gonium quadratum	AB774228				
Gonium viridistellatum	AB774224				
Volvox ferrisii	LC274877				
Volvox pergloba-tor	MG429691				

*Determined in this study.

Supplementary Table 9. Primers used for amplification and sequencing of the *FUS1* gene.

Primer name*	Sequence (5' to 3')	Forward (F) or Reverse (R)	Nucleotide positions**
PsFUS1_5UTRF1	GTAGAAAGACGGACAAGGACGGTG	F	14-37
PsFUS1_5UTRF2	TCGCGGATCTGACGACAGTGAGAG	F	38-61
PsFUS1_cFfirst	GCGGCCTTTCACGTTTGAAGTG	F	216-237
PsFUS1_cRfirst	CCAATGGGCCGTATGGATGGTTC	R	334-356
PsFUS1_cF1	ACGTCAATGGCAGCCCTTTC	F	503-522
PsFUS1_rGSP	ACTCTGCGCCGGCAGTACCAGATG	R	587-610
PsFUS1_cF2	ACAGACAATTTCACCAACTGG	F	637-657
PsFUS1_cR0	GCTTAGTTCGATAGTCACTACAGGAGAG	R	702-729
PsFUS1_cF25	AGCTGGTTTGCCAACTGGTGGATTG	F	727-751
PsFUS1_cF3	GAAATCCTGCCAGGCCCACTAAG	F	877-899
PsFUS1_cR1	CAAAATCCTGGACGCTTAGTG	R	893-913
PsFUS1_cF4	CCTTGCTATCTAGCAGGTTCATAC	F	1156-1179
PsFUS1_cR2	GAACAGCACTACAGATGTTGATGC	R	1201-1224
PsFUS1_cR3	TCCTGCACCATAGCCTATCACTCC	R	1585-1608
PsFUS1_cF6	GGGTAAGGCTACAAGATCGTTATGG	F	1676-1700
PsFUS1_cR4	GTAGTCCTTGATGCAAGACCTCG	R	1875-1897
PsFUS1_cF7	CACATTTTATGATGCTCATGACAAT	F	1989-2013
PsFUS1_cR5	ATAGCAACGCCCAGCATACAATCC	R	2140-2163
PsFUS1_cF8	TCAGTCGATATGACGTTGTGGGATATG	F	2272-2298
PsFUS1_cR6	GTTTGCTTCCCAGGAAGCCTCC	R	2361-2382
PsFUS1_cFlast	TGTGGATGAGGCCCAATCAGAAG	F	2628-2650
PsFUS1_cRlast	TAGGGTCGCTGAACATACAATCG	R	2726-2748
PsFUS1_3UTRR1	TGATCACGTGAAATGACCTGCTGC	R	2915-2938
PsFUS1_3UTRR2	GGCACACCCTTCACAAGACTTCTC	R	2985-3008

*All primers were designed in this study.

**The positions of *Pleodorina starrii FUS1* cDNA sequence (LC740508).

Supplementary Table 10. Specific primers of reverse transcription quantitative polymerase chain reaction (RT-qPCR) designed and used in the present study. All RT-qPCR reactions were performed with the following condition: 95℃ for 30 sec (Hold), 40 cycles of 95℃ for 5 sec, 60℃ for 30 sec (two-step PCR), and 95℃ for 15 sec, 60℃ for 30 sec, 95℃ for 15 sec (Dissociation).

**Primer name***	**Sequence (5' to 3')**	**Forward (F) or Reverse (R)**	**Amplicon length [bp]**	**Gene**
EF1L_qF2	CAAGAACATGATTTCCGGCG	F	111	*EF-1 like*
EF1L_qR2	ATCTCACCGGCCTTGTGGTC	R		
MID_qF2	TAGCTGCGAGTTACGAAATGGC	F	113	*MID*
MID_qR2	CCCGGAAATCTGGCTTCTG	R		
FUS1_qF3	ACAAATCTCGGAGCATCCCAC	F	127	*FUS1*
FUS1_qR3	TGGTGCTCCCCAAGAAGAAAG	R		
MTD1_qF3	CTGGACTACAGCAACCTGGTG	F	135	*MTD1*
MTD1_qR4	GTTTGGCAAGCCTGTGATG	R		
GCS1_qF2	CCTCCTGGGACCTGTCTTCA	F	129	*GCS1*
GCS1_qR2	GCTCAGCTCAACAGCGTCAC	R		

*All primers were designed in this study.

Supplementary References

1. Vurture, G. W., Sedlazeck, F. J., Nattestad, M., Underwood, C. J., Fang, H., Gurtowski, J. & Schatz, M. C. GenomeScope: fast reference-free genome profiling from short reads. *Bioinformatics* **33**, 2202-2204 (2017).
2. Nozaki, H., Mori, T., Misumi, O., Matsunaga, S. & Kuroiwa, T. Males evolved from the dominant isogametic mating type. *Curr. Biol.* **16**, 1018-1020 (2006).
3. Whelan, S. & Goldman, N. A general empirical model of protein evolution derived from multiple protein families using a maximum-likelihood approach. *Mol. Biol. Evol.* **18**, 691-699 (2001).
4. Kumar, S., Stecher, G., Li, M., Knyaz, C. & Tamura, K. MEGA X: Molecular Evolutionary Genetics Analysis across computing platforms. *Mol. Biol. Evol.* **35**, 1547-1549 (2018).
5. Hamaji, T., Mogi, Y., Ferris, P. J., Mori, T., Miyagishima, S., Kabeya, Y., Nishimura, Y., Toyoda, A., Noguchi, H., Fujiyama, A., Olson, B. J. S. C., Marriage, T. N., Nishii, I., Umen, J. G. & Nozaki, H. Sequence of the *Gonium pectorale* mating locus reveals a complex and dynamic history of changes in volvocine algal mating haplotypes. *G3* (*Bethesda*) **6**, 1179-1189 (2016).
6. Ferris, P. J., Woessner, J. P. & Goodenough, U. W. A sex recognition glycoprotein is encoded by the plus mating-type gene *fus1* of *Chlamydomonas reinhardtii*. *Mol. Biol. Cell* **7**, 1235-1248 (1996).
7. Yamashita, S., Yamamoto, K., Matsuzaki, R., Suzuki, S., Yamaguchi, H., Hirooka, S., Minakuchi, Y., Miyagishima, S. Y., Kawachi, M., Toyoda, A. & Nozaki, H. Genome sequencing of the multicellular alga *Astrephomene* provides insights into convergent evolution of germ-soma differentiation. *Sci. Rep.* **11**, 22231 (2021).
8. Hamaji, T., Kawai-Toyooka, H., Uchimura, H., Suzuki, M., Noguchi, H., Minakuchi, Y., Toyoda, A., Fujiyama, A., Miyagishima, S. Y., Umen, J. G. & Nozaki, H. Anisogamy evolved with a reduced sex-determining region in volvocine green algae. *Commun. Biol.* **1**, 17 (2018).
9. Yamamoto, K., Hamaji, T., Kawai-Toyooka, H., Matsuzaki, R., Takahashi, F., Nishimura, Y., Kawachi, M., Noguchi, H., Minakuchi, Y., Umen, J. G., Toyoda, A. & Nozaki, H. Three genomes in the algal genus *Volvox* reveal the fate of a haploid sex-determining region after a transition to homothallism. *Proc. Natl. Acad. Sci. U.S.A.* **118**, e2100712118 (2021).
10. Edgar, R. C. MUSCLE: multiple sequence alignment with high accuracy and high throughput. *Nucleic Acids Res.* **32**, 1792-1797 (2004).
11. Käll, L., Krogh, A. & Sonnhammer, E. L. L. Advantages of combined transmembrane topology and signal peptide prediction--the Phobius web server. *Nucleic Acids Res.* **35**,

429-432 (2007).
12. Ferris, P. J., Armbrust, E. V. & Goodenough, U. W. Genetic structure of the mating-type locus of *Chlamydomonas reinhardtii*. *Genetics* **160**, 181-200 (2002).
13. Hamaji, T., Ferris, P. J., Nishii, I. & Nozaki, H. Identification of the minus mating-type specific gene *mtd1* from *Gonium pectorale* (Volvocales, Chlorophyta). *J. Phycol*. **45**, 1310-1314 (2009).
14. Letunic, I & Bork, P. 20 years of the SMART protein domain annotation resource. *Nucleic Acids Res*. **46**, 493-496 (2017).
15. Letunic, I., Khedkar, S. & Bork, P. SMART: recent updates, new developments and status in 2020. *Nucleic Acids Res*. **4**9, 458-460 (2020).
16. Chauhan, J. S., Rao, A. & Raghava, G. P. S. In silico Platform for Prediction of N-, O- and C-Glycosites in Eukaryotic Protein Sequences. *PLoS One* **8**, e67008 (2013).
17. Liu, Y., Tewari, R., Ning, J., Blagborough, A. M., Garbom, S., Pei, J., Grishin, N. V., Steele, R. E., Sinden, R. E., Snell, W. J. & Billker, O. The conserved plant sterility gene HAP2 functions after attachment of fusogenic membranes in *Chlamydomonas* and *Plasmodium* gametes. *Genes Dev*. **22**, 1051-1068 (2008).
18. Kawai-Toyooka, H., Mori, T., Hamaji, T., Suzuki, M., Olson, B. J., Uemura, T., Ueda, T., Nakano, A., Toyoda, A., Fujiyama, A. & Nozaki, H. Sex-specific posttranslational regulation of the gamete fusogen *GCS1* in the isogamous volvocine alga *Gonium pectorale*. *Eukaryot*. *Cell* **13**, 648-656 (2014).
19. Prochnik, S. E., Umen, J., Nedelcu, A. M., Hallmann, A., Miller, S. M., Nishii, I., Ferris, P., Kuo, A., Mitros, T., Fritz-Laylin, L. K., Hellsten, U., Chapman, J., Simakov, O., Rensing, S. A., Terry, A., Pangilinan, J., Kapitonov, V., Jurka, J., Salamov, A., Shapiro, H., Schmutz, J., Grimwood, J., Lindquist, E., Lucas, S., Grigoriev, I. V., Schmitt, R., Kirk, D. & Rokhsar, D. S. Genomic analysis of organismal complexity in the multicellular green alga *Volvox carteri*. *Science* **329**, 223-226 (2010).
20. Blum, M., Chang, H. Y., Chuguransky, S., Grego, T., Kandasaamy, S., Mitchell, A., Nuka, G., Paysan-Lafosse, T., Qureshi, M., Raj, S., Richardson, L., Salazar, G. A., Williams, L., Bork, P., Bridge, A., Gough, J., Haft, D. H., Letunic, I., Marchler-Bauer, A., Mi, H., Natale, D. A., Necci, M., Orengo, C. A., Pandurangan, A. P., Rivoire, C., Sigrist, C. J. A., Sillitoe, I., Thanki, N., Thomas, P. D., Tosatto, S. C. E., Wu, C. H., Bateman, A. & Finn, R. D. The InterPro protein families and domains database: 20 years on. *Nucleic Acids Res*. **49**, 344-354 (2021).
21. Lindsey, C. R., Rosenzweig, F. & Herron, M. D. Phylotranscriptomics points to multiple independent origins of multicellularity and cellular differentiation in the volvocine algae. *BMC Biol.* **19**:182 (2021).
22. Renner, S. S. The relative and absolute frequencies of angiosperm sexual systems: Dioecy, monoecy, gynodioecy, and an updated online database. *Am. J. Bot.* **101**, 1588-

1596 (2014).

23. Bachtrog, D., Mank, J. E., Peichel, C. L., Kirkpatrick, M., Otto, S. P., Ashman, T. L., Hahn, M. W., Kitano, J., Mayrose, I., Ming, R., Perrin, N., Ross, L., Valenzuela, N., Vamosi, J. C. & Tree of Sex Consortium. Sex determination: why so many ways of doing it? *PLoS Biol*. **12**, e1001899 (2014).
24. Kojis, B. L. & Quinn, N. J. Aspects of sexual reproduction and larval development in the shallow-water hermatypic coral, *Goniastrea australensis* (Edwards and Haime, 1857). *Bull. Mar. Sci.* **31**, 558–573 (1981).
25. Ward, S. & Carrel, J. S. Fertilization and sperm competition in the nematode *Caenorhabditis elegans*. *Dev. Biol*. **73**, 304–321 (1979).
26. Jolls, C. L. & Chenier, T. C. Gynodioecy in *Silene Vulgaris* (Caryophyllaceae): Progeny success, experimental design, and maternal effects. *Am. J. Bot*. **76**, 1360-1367 (1989).
27. Molano-Flores, B. Pollination biology and flower visitors of the gynodioecious species *Lobelia spicata* Lam. (Campanulaceae). *J. Torrey Bot. Soc*. **129**, 187-193 (2002).
28. Wang, J., Na, J. K., Yu, Q., Gschwend, A. R., Han, J., Zeng, F., Aryal, R., VanBuren, R., Murray, J. E., Zhang, W., Navajas-Pérez, R., Feltus, F. A., Lemke, C., Tong, E. J., Chen, C., Wai, C. M., Singh, R., Wang, M. L., Min, X. J., Alam, M., Charlesworth, D., Moore, P., Jiang, J., Paterson, A. & Ming, R. Sequencing papaya X and Y h chromosomes reveals molecular basis of incipient sex chromosome evolution. *Proc. Natl. Acad. Sci. U.S.A.* **109**, 13710-13715 (2012).
29. Kanzaki, N., Kiontke, K., Tanaka, R., Hirooka, Y., Schwarz, A., Müller-Reichert, T., Chaudhuri, J. & Pires-daSilva, A. Description of two three-gendered nematode species in the new genus *Auanema* (Rhabditina) that are models for reproductive mode evolution. *Sci. Rep*. **7**, 11135 (2017).
30. Bold, H. C., Alexopoulos, C. J. & Delevoryas, T. *Morphology of Plants and Fungi. 5th Edn.* Harper & Row, New York (1987).
31. Takahashi, K., Kawai-Toyooka, H., Ootsuki, R., Hamaji, T., Tsuchikane, Y., Sekimoto, H., Higashiyama, T. & Nozaki, H. Three sex phenotypes in a haploid algal species give insights into the evolutionary transition to a self-compatible mating system. *Evolution* **75**, 2984-2993 (2021).
32. Roy, S. W. Digest: Three sexes from two loci in one genome: A haploid alga expands the diversity of trioecious species. *Evolution* **75**, 3002-3003 (2021).
33. Flynn, J. M., Hubley, R., Goubert, C., Rosen, J., Clark, A. G., Feschotte, C. & Smit, A. F. RepeatModeler2 for automated genomic discovery of transposable element families. *Proc. Natl. Acad. Sci. U.S.A.* **117**, 9451-9457 (2020).
34. Manni, M., Berkeley, M. R., Seppey, M., Simão, F. A. & Zdobnov, E. M. BUSCO update: Novel and streamlined workflows along with broader and deeper phylogenetic coverage for scoring of eukaryotic, prokaryotic, and viral genomes. *Mol. Biol. Evol*. **38**,

4647-4654 (2021).

35. Nielsen, R. & Yang, Z. Likelihood models for detecting positively selected amino acid sites and applications to the HIV-1 envelope gene. *Genetics* **148**, 929–936 (1998).
36. Yang, Z., Wong, W. S. W. & Nielsen, R. Bayes empirical Bayes inference of amino acid sites under positive selection. *Mol. Biol. Evol.* **22**, 1107–1118 (2005).
37. Yang, Z., Nielsen, R., Goldman, N. & Pedersen, A.-M. K. Codon-substitution models for heterogeneous selection pressure at amino acid sites. *Genetics* **155**, 431–449 (2000).
38. Yang, Z. PAML 4: phylogenetic analysis by maximum likelihood. *Mol. Biol. Evol.* **24**, 1586-1591 (2007).
39. Ferris, P., Olson, B. J., De Hoff, P. L., Douglass, S., Casero, D., Prochnik, S., Geng, S., Rai, R., Grimwood, J., Schmutz, J., Nishii, I., Hamaji, T., Nozaki, H., Pellegrini, M. & Umen, J. G. Evolution of an expanded sex-determining locus in *Volvox*. *Science* **328**, 351-354 (2010).
