# Supplementary material for: Reorganization of the ancestral sex-determining regions during the evolution of trioecy in Pleodorina starrii
Source: Commun Biol. 2023 Jun 9;6:590. doi: 10.1038/s42003-023-04949-1 (PMC10256686; doi:10.1038/s42003-023-04949-1)
Supplement: Supplementary file 3 — Description of Additional Supplementary Files [file 42003_2023_4949_MOESM3_ESM.pdf]

Description of Additional Supplementary Files

File name: Supplementary Data 1
Description: Data analysis for RT-qPCR of four sex-related genes (Fig. 4).

File name: Supplementary Data 2
Description: Data analyses for molecular evolutionary analyses of gamelotogs of seven volvocine species (Supplementary Fig. 5).
