# Supplementary material for: Reorganization of the ancestral sex-determining regions during the evolution of trioecy in Pleodorina starrii
Source: Commun Biol. 2023 Jun 9;6:590. doi: 10.1038/s42003-023-04949-1 (PMC10256686; doi:10.1038/s42003-023-04949-1)
Supplement: Supplementary file 6 — Reporting Summary [file 42003_2023_4949_MOESM6_ESM.pdf]

nature portfolio

Corresponding author(s): Hisayoshi Nozaki

Last updated by author(s): May 17, 2023

Reporting Summary

Nature Portfolio wishes to improve the reproducibility of the work that we publish. This form provides structure for consistency and transparency in reporting. For further information on Nature Portfolio policies, see our Editorial Policies and the Editorial Policy Checklist.

Statistics

For all statistical analyses, confirm that the following items are present in the figure legend, table legend, main text, or Methods section.

n/a	Confirmed	
☐	☒	The exact sample size (*n*) for each experimental group/condition, given as a discrete number and unit of measurement
☐	☒	A statement on whether measurements were taken from distinct samples or whether the same sample was measured repeatedly
☐	☒	The statistical test(s) used AND whether they are one- or two-sided<br>*Only common tests should be described solely by name; describe more complex techniques in the Methods section.*
☒	☐	A description of all covariates tested
☒	☐	A description of any assumptions or corrections, such as tests of normality and adjustment for multiple comparisons
☐	☒	A full description of the statistical parameters including central tendency (e.g. means) or other basic estimates (e.g. regression coefficient) AND variation (e.g. standard deviation) or associated estimates of uncertainty (e.g. confidence intervals)
☒	☐	For null hypothesis testing, the test statistic (e.g. *F*, *t*, *r*) with confidence intervals, effect sizes, degrees of freedom and *P* value noted<br>*Give P values as exact values whenever suitable.*
☐	☒	For Bayesian analysis, information on the choice of priors and Markov chain Monte Carlo settings
☒	☐	For hierarchical and complex designs, identification of the appropriate level for tests and full reporting of outcomes
☒	☐	Estimates of effect sizes (e.g. Cohen's *d*, Pearson's *r*), indicating how they were calculated

Our web collection on statistics for biologists contains articles on many of the points above.

Software and code

Policy information about availability of computer code

Data collection	Long read sequencing was obtained using the PacBio Sequel II instrument (Pacific Biosciences, Menlo Park, CA, USA) and SMRTbell® Express Template Prep Kit 2.0 (Pacific Biosciences). Short read sequencing was obtained using the Illumina NovaSeq 6000 instrument (150 bp × 2 library; Illumina, San Diego, CA, USA) with the NEBNEXT® Ultra DNA Library Prep Kit for Illumina (New England Biolabs, Ipswich, MA, USA).
Data analysis	All softwares used for analyzing the nucleotide sequence data have been descried in the main manuscript and supplementary information.

For manuscripts utilizing custom algorithms or software that are central to the research but not yet described in published literature, software must be made available to editors and reviewers. We strongly encourage code deposition in a community repository (e.g. GitHub). See the Nature Portfolio guidelines for submitting code & software for further information.

Data

Policy information about availability of data

All manuscripts must include a data availability statement. This statement should provide the following information, where applicable:

- Accession codes, unique identifiers, or web links for publicly available datasets
- A description of any restrictions on data availability
- For clinical datasets or third party data, please ensure that the statement adheres to our policy

Raw reads of genome and RNA-seq data and genome assemblies with annotations data have been deposited in DNA Data Bank of Japan (DDBJ)/European Molecular Biology Laboratory (EMBL)/GenBank (DDBJ Sequence Read Archive [DRA]: DRA015329; whole-genome assemblies with annotations: BRXT01000001through

BRXT01000242 [P. starrii NIES-1363 male phenotype], BRXV01000001 through BRXV01000288 [P. starrii NIES-4481 female phenotype], and BRXU01000001 through BRXU01000095 [P. starrii NIES-4479 bisexual phenotype]]). SDR genome sequences harboring rearranged domains with fully sex-linked genes in P. starrii male, female and bisexual phenotypes, are available under accession numbers SMU11264 through SMU11275. All other study data are included in the article and Supplementary Information.

Human research participants

Policy information about studies involving human research participants and Sex and Gender in Research.

Reporting on sex and gender	The study did not involve human research participants and sex and gender in research.
Population characteristics	NA
Recruitment	NA
Ethics oversight	NA

Note that full information on the approval of the study protocol must also be provided in the manuscript.

Field-specific reporting

Please select the one below that is the best fit for your research. If you are not sure, read the appropriate sections before making your selection.

☒ Life sciences ☐ Behavioural & social sciences ☐ Ecological, evolutionary & environmental sciences

For a reference copy of the document with all sections, see nature.com/documents/nr-reporting-summary-flat.pdf

Life sciences study design

All studies must disclose on these points even when the disclosure is negative.

Sample size	NA
Data exclusions	No data were excluded from the analyses.
Replication	Expression data from RT-qPCR for each sex-related genes (Figure 4) were obtained from three independent biological samples to confirm reproducibility.
Randomization	This is relevant to estimations of bootstrap values of maximum likelihood analysis (Figures 2B, 2C, 2E and 3C; Supplementary Figure 6). Bootstrap re-samplings of the sequence data were randomly performed by the software MEGA.
Blinding	NA

Reporting for specific materials, systems and methods

We require information from authors about some types of materials, experimental systems and methods used in many studies. Here, indicate whether each material, system or method listed is relevant to your study. If you are not sure if a list item applies to your research, read the appropriate section before selecting a response.

Materials & experimental systems

n/a	Involved in the study	
☒	☐	Antibodies
☒	☐	Eukaryotic cell lines
☒	☐	Palaeontology and archaeology
☒	☐	Animals and other organisms
☒	☐	Clinical data
☒	☐	Dual use research of concern

Methods

n/a	Involved in the study	
☒	☐	ChIP-seq
☒	☐	Flow cytometry
☒	☐	MRI-based neuroimaging
