# Supplementary material for: Reorganization of the ancestral sex-determining regions during the evolution of trioecy in Pleodorina starrii
Source: Commun Biol. 2023 Jun 9;6:590. doi: 10.1038/s42003-023-04949-1 (PMC10256686; doi:10.1038/s42003-023-04949-1)
Supplement: Supplementary file 1 — Peer Review File [file 42003_2023_4949_MOESM1_ESM.pdf]

nature portfolio

Peer Review File

Reorganization of the ancestral sex-determining regions during the evolution of trioecy in Pleodorina starrii

Open Access This file is licensed under a Creative Commons Attribution 4.0 International License, which permits use, sharing, adaptation, distribution and reproduction in any medium or format, as long as you give appropriate credit to the original author(s) and the source, provide a link to the Creative Commons license, and indicate if changes were made. In the cases where the authors are anonymous, such as is the case for the reports of anonymous peer reviewers, author attribution should be to 'Anonymous Referee' followed by a clear attribution to the source work. The images or other third party material in this file are included in the article's Creative Commons license, unless indicated otherwise in a credit line to the material. If material is not included in the article's Creative Commons license and your intended use is not permitted by statutory regulation or exceeds the permitted use, you will need to obtain permission directly from the copyright holder. To view a copy of this license, visit http://creativecommons.org/licenses/by/4.0/.

Reviewers' comments:

Reviewer #1 (Remarks to the Author):

This is an interesting study reporting the sequencing and comparative analysis of the male, female and bisex genomes in Pleodorina strarrii green algae that has sex determination at the haploid stage of its lifecycle. They report that the sex-determining region (SDR) with suppressed recombination are very small, ~150kb and that bisexuals have the same SDR as the males. Surprisingly, the female SDR lacks the key 'female gene' FUS1 that is located on an autosome. As such, the female SDR appears to play no role in sex determination. Instead, FUS1 is suppressed by MID located in male SDR, but this suppression is suppressed by the bisexual factor (BF) located somewhere on an autosome.

The story presented is interesting and worth publishing, but I miss a bit of generalisation in the discussion. The described sex-determination in P. starrii reminds me of what happens with sex-determining genes during sex chromosome turnover events in diploid organisms. Perhaps we see a sex chromosome turnover in making in this algal species and it would be great to add a bit of discussion along these lines and discuss the parallels with diploid organisms (e.g. look at sex determination in Musca domestica where there are several sex-determining factors suppressing each other).

The results are clearly presented, but the writing deserves much improvement. I made some comments along these lines below, but I think the text has to be read and corrected very thoroughly. This is not always about English. Often the problems are in the way they formulate and state things, as I explain below.

Fig1 top left corner: "l" missing in "unisexua"

Line 30-31: "…trioecy in haploid algae and fungi… in algae species Pleodorina…" not sure what "fungi" is doing in this sentence given the statement is about algal species P.starrii.

Line 31: change "green algae species" to "green algal species"

Line 58. "In haploid organisms such as algae and fungi" – not all algae and fungi are haploid.

Line 89. Please specify here which version of PacBio was used – HiFi or older technologies.

Lines 105-118. This paragraph reads as part of introduction rather than the results section.

Line 109. Incorrect statement: "sex-specific (fully sex-linked)". The same applies to line 237. Better change to "sex-specific (present in one sex only)". Fully sex-linked does not mean sex-specific because gametologs present in both sexes (or mating types) can also be fully sex-linked. For example, in more 'conventional' sex-determination in mammals there are many Y-linked (fully sex-linked) genes that have gametologs on the X-chromosome.

Line 117. Orthologs or paralogs? Please check.

Line 139. More informative to write here "contained MID and its two dysfunctional paralogs" as "three paralogs" suggests that there are three functional copies of MID

Line 146. Fix typo in "acordi_ng"

Lines 148-150 and Suppl figure 5. Synonymous divergence between gametologs in P. starrii is quite small, implying a recent origin of this sex-determining region (and cessation of recombination in the region). It would be good to add some discussion of the origin of SDR in P.starrii, and perhaps estimate SDR age, given the dS for the gametologs in this region.

Line 289 (and possibly elsewhere in the paper): Based on the data presented, I do not see "drastic reorganisation of the genome". Yes, FUS1 was moved to an autosome and a bisexual factor arose elsewhere, but this can hardly qualify as a "drastic reorganisation of the genome".

Reviewer #2 (Remarks to the Author):

Brief summary of the manuscript.
This manuscript details a genomic analysis of the content and organisation of sex-determining regions (SDR) in the trioecious green algae Pleodorina starrii. The analysis is based on the comparison of novel genome assemblies obtained separately from each of the three sexual phenotypes (females, males and bisexual). The results show that the male and bisexual genotypes have nearly identical SDR, and diverge from that of the female SDR. A striking observation is that the FUS1 gene, which is associated with the female phenotype in other volvocine algae is absent from the female SDR, and instead acquired an autosomal chromosomal location. qPCR analysis shows that its expression is still restricted to the female colonies, suggesting an unknown regulatory mechanism.

Overall impression of the work.
Overall, the major claims of the paper relate to the genomic basis of the control of a highly unusual reproductive system (trioecy). The results are highly novel, they seem to be sound, and they advance our understanding of this fascinating genetic system. They will appeal to a broad community of researchers interested in the molecular control of reproductive phenotypes and the evolution of sex chromosomes, who will be inspired by the unexpected finding that trioecy is associated with translocation of one of the main sex-determining genes to an autosome.

Specific comments, with recommendations for addressing each comment.
1- I found the manuscript hard to follow at many places. In particular, several key pieces of information are either fully lacking or are given after they would actually be needed. For example, it still remains obscure to me what some of the genes studied are actually doing and why they are worth focusing on (why focusing on MTD1, PSMT, GCS1/HAP2 ? is this a comprehensive list of all sex-specific genes, and if not what were the reasons to focus on them specifically ?).

2- Along the same line, the detailed description of the duplications of these genes is lengthy and poorly focused. As such, it remains purely descriptive, and in the absence of insight about the eventual consequences of these duplications it is hard to evaluate what to make of this information.

3- The genetic analysis of Takahashi et al. 2021 is the main foundation for this paper, but it is not presented in sufficient details for the readers to fully distinguish between what they firmly established and
what remained unsure. Figure 1 is helpful with this regard, but it is not sufficiently explained in the introduction. Furthermore, some key insight from this previous study are too succintly presented. In particular, the action of the « bisexual factor » is essential, but it is first presented on line 256 at the

middle of the discussion, and not at all mentioned in the introduction.

4- the evolution of the female SDR to resemble the genomic background in terms of GC and repeat content is a striking result that could be given more prominence. I understand that the authors currently have no explanation, but it would be helpful to at least speculate on the processes by which SDRs in haploid systems tend to evolve in a different manner as compared to the rest of the genome. There is a rich and active literature on these fascinating systems (e.g. 10.1016/j.tplants.2018.06.005) that could be acknowldeged.

5- I was also a bit frustrated by the lack of speculation about the mechanistic consequences of the translocation of FUS1 to an autosome. What kind of regulation does that exactly entail ? The text tends to remain descriptive, by documenting the genomic changes that occurred. Each of these changes are interesting in their own right, but ideally one would like to understand in a more precise manner the role they played in the evolution and maintenance of trioecy. The discussion ends with the proposition that a « drastic reorganization of the genome » was necessary for the evolution of trioecy. At this stage, I am not convinced by this claim, because one could make the argument that maybe just one of these changes was indeed sufficient, and all the others are just neutral changes that accumulated later on, with no functional consequences for the mating system. Overall, I think that the link between the observed genomic changes and the emergence of this very unusual mating system needs to be improved in the manuscript.

6. The claim that « All dN/dS values … were <1.0 indicat[es] no positively selected gametologs » is incorrect. In fact, very few positively selected genes exhibit dN/dS>1, because gene are typically composed of codons with a variety of evolutionary constraints. Some codons can exhibit dN/dS>1, while some others along the same gene exhibit strong functional constraints (and hence dN/dS<<1), such that overall it is rare to observe dN/dS>1 at the complete gene level. Ideally this kind of analyses should thus be performed at the codon rather than at the gene level, but I doubt whether sufficient power will remain unless orthologs from a large number of other species are also included.

Our responses to the reviewers’ comments have been described below# in red.

Sincerely,

Dr. Hisayoshi Nozaki

#
Reviewers' comments:

Reviewer #1 (Remarks to the Author):

This is an interesting study reporting the sequencing and comparative analysis of the male, female and bisex genomes in Pleodorina strarrii green algae that has sex determination at the haploid stage of its lifecycle. They report that the sex-determining region (SDR) with suppressed recombination are very small, ~150kb and that bisexuals have the same SDR as the males. Surprisingly, the female SDR lacks the key ‘female gene’ FUS1 that is located on an autosome. As such, the female SDR appears to play no role in sex determination. Instead, FUS1 is suppressed by MID located in male SDR, but this suppression is suppressed by the bisexual factor (BF) located somewhere on an autosome.

The story presented is interesting and worth publishing, but I miss a bit of generalisation in the discussion. The described sex-determination in P. starrii reminds me of what happens with sex-determining genes during sex chromosome turnover events in diploid organisms. Perhaps we see a sex chromosome turnover in making in this algal species and it would be great to add a bit of discussion along these lines and discuss the parallels with diploid organisms (e.g. look at sex determination in Musca domestica where there are several sex-determining factors suppressing each other).
Response: Based on the comments, we have added new sentences of a discussion along these lines to the end of the first paragraph of the Discussion section of the revised manuscript (*<u>**lines 373-381**</u> of marked up version<u>**).**</u>
*Thus, the sex-determination in *P. starrii* is probably based on the modification of the typical heterothallic system with male and female SDRs by the acquisition of two autosomal sex genes, *FUS1* and "BF" (Fig. 1). This situation is similar to the polygenic or multifactorial sex determination in the house fly *Musca*

domestica [24,25]. *M. domestica* has X and Y sex chromosomes. In the ancestral state, the sex was determined by the presence or absence of a male-determining locus (M factor) on the Y chromosome[25]. Thus, typical males are XY^M and females are XX. However, males with the M factor on the autosomes (A^M) or the X chromosome (X^M) can be found in natural populations[25]. The stable frequency of A^M found in natural populations of *M. domestica* over 30 years suggests that the polygenic sex determination system of the house fly is not in transition[26]. Long-term field data on the three sex phenotypes of *P. starrii* are expected to determine whether or not the trioecy represents a transient state.

The results are clearly presented, but the writing deserves much improvement. I made some comments along these lines below, but I think the text has to be read and corrected very thoroughly. This is not always about English. Often the problems are in the way they formulate and state things, as I explain below.
Response: In addition to revisions suggested by the comments kindly made by the reviewer, we have read and corrected the manuscript very thoroughly. The figures for SDRs of *Pleodorina starriii* have been much improved with revised annotations of the SDR genes in the revised manuscript (Fig. 2A; Supplementary Figs. 2, 3; pages 4-5 of the revised main manuscript).

Fig1 top left corner: “l” missing in “unisexua”
Response: Thank you very much for your very critical comment. The spelling in Figure 1 has been revised.

Line 30-31: “…trioecy in haploid algae and fungi… in algae species Pleodorina…” not sure what “fungi” is doing in this sentence given the statement is about algal species P.starrii.
Response: Based on the comments, "trioecy in haploid algae and fungi" has been revised to "trioecy in haploid organisms" in the revised marked up manuscript with tracking revisions (**line 30**).

Line 31: change “green algae species” to “green algal species”
Response: Revised as suggested.

Line 58. “In haploid organisms such as algae and fungi” – not all algae and

fungi are haploid.

Response: Based on the comment, the sentence has been revised as “In haploid algae and fungi” (**line 63** of the revised marked up manuscript with tracking revisions).

Line 89. Please specify here which version of PacBio was used – HiFi or older technologies.

Response: As described in the Materials and Methods section, we used PacBio Sequel II/SMRT technology, which enables High-Fidelity (HiFi) Long Reads <https://dnatech.genomecenter.ucdavis.edu/pacbio-library-prep-sequencing/ >. The version of PacBio and SMRT were already described in in the Materials and Methods section, and (see “Materials and Methods”) was already inserted in the posterior end of the sentence. Thus, “high-fidelity” has been inserted just before “PacBio” in the sentence (**line121** of the revised marked up manuscript with tracking revisions).

Lines 105-118. This paragraph reads as part of introduction rather than the results section.

Response: Based on the comment, this paragraph has been moved to the third paragraph of the Introduction section in the revised manuscript (**lines 70-93** of the revised marked up manuscript with tracking revisions).

Line 109. Incorrect statement: “sex-specific (fully sex-linked)”. The same applies to line 237. Better change to “sex-specific (present in one sex only)”. Fully sex-linked does not mean sex-specific because gametologs present in both sexes (or mating types) can also be fully sex-linked. For example, in more ‘conventional’ sex-determination in mammals there are many Y-linked (fully sex-linked) genes that have gametologs on the X-chromosome.

Response: Based on the comment, “fully sex-linked gene” has been removed and “sex-specific gene” has been used throughout the manuscript of the revised version.

Line 117. Orthologs or paralogs? Please check.

Response: I have checked all such genes in the published references. All such genes in autosomal region are single-copy in each heterothallic species. Thus, revision has not been done.

Line 139. More informative to write here “contained MID and its two dysfunctional paralogs” as “three paralogs” suggests that there are three functional copies of MID

Response: Based on the comments, more detailed description of the three paralogs of MID has been described in the revised manuscript (***lines 221-223** in the revised marked up main manuscript with tracking revisions).

*One of the three *MID* paralogs was functional and encoded the full MID protein sequence (163 amino acids) whereas the other two were pseudogenes encoding only 75 or 120 amino acids (Supplementary Fig. 4).

Line 146. Fix typo in “acordi_ng”

Response: The typo has been revised as suggested.

Lines 148-150 and Suppl figure 5. Synonymous divergence between gametologs in P. starrii is quite small, implying a recent origin of this sex-determining region (and cessation of recombination in the region). It would be good to add some discussion of the origin of SDR in P.starrii, and perhaps estimate SDR age, given the dS for the gametologs in this region.

Response: Based on the comment, some discussion of the recent origin and/or recent gene conversion between part (inverted regions) of male and female SDRs in *P. starrii* has been described in the revised manuscript (***lines 161-163**, and ****lines 234-242** in the marked up file with tracking revisions).

*, with three inverted sequences (measuring approximately half the SDR in total) within the male and female SDRs (Fig. 2A; Supplementary Fig. 2).

** Since the synonymous divergence between gametologs in *P. starrii* is quite low and the majority of the gametologs in *P. starrii* are localized within the inverted regions (Supplementary Figs. 2, 5), this suggests a recent origin of the *P. starrii* SDR or a recent gene conversion (inverted regions) between male and female SDRs.

Line 289 (and possibly elsewhere in the paper): Based on the data presented, I do not see “drastic reorganisation of the genome”. Yes, FUS1 was moved to an autosome and a bisexual factor arose elsewhere, but this can hardly qualify as a “drastic reorganisation of the genome”.

Response: Based on the comment, “drastic” has been removed throughout the

manuscript of the revised version.

Reviewer #2 (Remarks to the Author):

Brief summary of the manuscript.
This manuscript details a genomic analysis of the content and organisation of sex-determining regions (SDR) in the trioecious green algae Pleodorina starrii. The analysis is based on the comparison of novel genome assemblies obtained separately from each of the three sexual phenotypes (females, males and bisexual). The results show that the male and bisexual genotypes have nearly identical SDR, and diverge from that of the female SDR. A striking observation is that the FUS1 gene, which is associated with the female phenotype in other volvocine algae is absent from the female SDR, and instead acquired an autosomal chromosomal location. qPCR analysis shows that its expression is still restricted to the female colonies, suggesting an unknown regulatory mechanism.

Overall impression of the work.
Overall, the major claims of the paper relate to the genomic basis of the control of a highly unusual reproductive system (trioecy). The results are highly novel, they seem to be sound, and they advance our understanding of this fascinating genetic system. They will appeal to a broad community of researchers interested in the molecular control of reproductive phenotypes and the evolution of sex chromosomes, who will be inspired by the unexpected finding that trioecy is associated with translocation of one of the main sex-determining genes to an autosome.

Specific comments, with recommendations for addressing each comment.
1- I found the manuscript hard to follow at many places. In particular, several key pieces of information are either fully lacking or are given after they would actually be needed. For example, it still remains obscure to me what some of the genes studied are actually doing and why they are worth focusing on (why focusing on MTD1, PSMT, GCS1/HAP2 ? is this a comprehensive list of all sex-specific genes, and if not what were the reasons to focus on them specifically ?).

Response: Why we focus MTD1 and GCS1 is based on the previous studies that suggest that these genes are important in male gametogenesis and gamete fusion. PSMT genes are genes that are localized within the SDR but lack homology to other genes to be annotated as “unknown function.” Based on the comments. Some explanations for these sex-related genes have been described in the revised manuscript (***lines 270-275** in the marked up file with tracking revisions).

* We also examined two conserved sex-related genes, *MTD1*[12,13] and *GCS1*/*HAP2*[22], in *P*. *starrii*. *MTD1* is considered to regulate *MID* in *Chlamydomonas reinhardti* and is found in various volvocine species. *MTD1* is male-specific (present in male SDR) in the oogamous volvocine species *Volvox reticuliferus* or found in the PAR adjacent to the SDR in *Y*. *unicocca* and *Eudorina* sp[12,13].

2- Along the same line, the detailed description of the duplications of these genes is lengthy and poorly focused. As such, it remains purely descriptive, and in the absence of insight about the eventual consequences of these duplications it is hard to evaluate what to make of this information.

Response: Duplication or paralogous expansion of *GCS1/HAP2* and *MID* are very rare in the eukaryotes and volvocine green algae, respectively. Thus, detailed information of these genes was described in the Results section in the original manuscript. Discussion such paralogous expansion was described in the Discussion section of the revised manuscript. Thus, the discussion has been expanded and detailed in the revised manuscript (***lines 404-408** in the marked up file with tracking revisions).

*Such mechanisms may play a role in the differential transcriptional regulation of *MID*, *GCS1*, and their downstream sex-related genes among the three sex phenotypes in *P*. *starrii* (Fig. 1). Thus, paralogous expansion of *MID* and *GCS1* may have underpinned the origin of trioecy in *P. starrii*.

3- The genetic analysis of Takahashi et al. 2021 is the main foundation for this paper, but it is not presented in sufficient details for the readers to fully distinguish between what they firmly established and
what remained unsure. Figure 1 is helpful with this regard, but it is not sufficiently explained in the introduction. Furthermore, some key insight from this previous study are too succintly presented. In particular, the action of the

« bisexual factor » is essential, but it is first presented on line 256 at the middle of the discussion, and not at all mentioned in the introduction.

Response: Based on the comments, some explanation for the sex-determining system of the trioecious *P. starrrii* has been added in the Introduction section of the revised manuscript (*__lines 101-104__ and **__lines 107-108__ in the marked up file with tracking revisions).

*: SDR on the sex chromosome and bisexual factor (BF) on the autosome (Fig. 1). The male and bisexual phenotypes have the identical "male SDR" whereas the female phenotype harbors the female SDR. BF plays a role in determining the bisexual phenotype in the presence of the male SDR (Fig. 1).

** However, molecular genetic features of the SDR and BF in *P. starrii* have remained unresolved.

4- the evolution of the female SDR to resemble the genomic background in terms of GC and repeat content is a striking result that could be given more prominence. I understand that the authors currently have no explanation, but it would be helpful to at least speculate on the processes by which SDRs in haploid systems tend to evolve in a different manner as compared to the rest of the genome. There is a rich and active literature on these fascinating systems (e.g. 10.1016/j.tplants.2018.06.005) that could be acknowldeged.

Response: Based on the suggestion and the literature, a speculative discussion has been added to the Discussion section to make the results more striking in the revised manuscript (*__lines 413-415__ in the file marked up with tracking revisions).

* However, one could only speculate that during the evolution of trioecy, the female SDR may have lost the processes by which SDRs in haploid systems tend to evolve differently from the rest of the genome[9].

5- I was also a bit frustrated by the lack of speculation about the mechanistic consequences of the translocation of FUS1 to an autosome. What kind of regulation does that exactly entail ? The text tends to remain descriptive, by documenting the genomic changes that occurred. Each of these changes are interesting in their own right, but ideally one would like to understand in a more precise manner the role they played in the evolution and maintenance of trioecy. The discussion ends with the proposition that a « drastic reorganization of the genome » was necessary for the evolution of trioecy. At this stage, I am

not convinced by this claim, because one could make the argument that maybe just one of these changes was indeed sufficient, and all the others are just neutral changes that accumulated later on, with no functional consequences for the mating system. Overall, I think that the link between the observed genomic changes and the emergence of this very unusual mating system needs to be improved in the manuscript.
Response: Based on the comment, a paragraph of such discussion has been added to the end of the Discussion section of the revised manuscript (***lines 452-463** in the file marked up with tracking revisions).
*Our study resolved very unique genomic features of the trioecious species *Pleodrina starrii*, including the transposition of *FUS1* from the female SDR to the autosomal region, paralogous expansions of *MID* and *GCS1*, and the low GC and low repeat rich female *SDR*. As discussed above, the transposition of *FUS1* to the autosomal region of the three sex phenotypes is the fundamental genetic basis for harboring the female gene *FUS1* and the male gene *MID* in a single haploid genome of the bisexual phenotype. However, the specific role of other genomic features in the evolution and maintenance of trioecy is unknown. In addition, our previous genetic analysis suggested a putative factor (BF) that determines the bisexual phenotype in the presence of the male SDR (Fig. 1). One could speculate that only one of these was actually sufficient, and that all the others are just neutral changes that accumulated later without functional consequences for the mating system.

6. The claim that « All dN/dS values … were <1.0 indicat[es] no positively selected gametologs » is incorrect. In fact, very few positively selected genes exhibit dN/dS>1, because gene are typically composed of codons with a variety of evolutionary constraints. Some codons can exhibit dN/dS>1, while some others along the same gene exhibit strong functional constraints (and hence dN/dS<<1), such that overall it is rare to observe dN/dS>1 at the complete gene level. Ideally this kind of analyses should thus be performed at the codon rather than at the gene level, but I doubt whether sufficient power will remain unless orthologs from a large number of other species are also included.
Response: Except for *LEU1S*, all gametologs in *P. starrii* lack homologous genes in other volvocine algae. Thus, the codon-based positive selection was examined in only *LEU1S*, suggesting no positive selection in *LEU1S*. Such data (Supplementary Table 4 of the revised version) has been prepared and a

discussion has been described in the Results section of the revised manuscript (***lines 234-238** in the file marked up with tracking revisions).
*Except for *LEU1S*, all gametologs in *P. starrii* lack homologous genes in other volvocine algae. Such gametologs in *P. starrii* cannot be aligned with homologous sequences from other volvocine green algae. Thus, the codon-based test of positive selection was only examined in *LEU1S*, suggesting no positive selection in *LEU1S* (Supplementary Table 4).

** See the Nature Portfolio author and referees' website at www.nature.com/authors for information about policies, services and author benefits

Communications Biology is committed to improving transparency in authorship. As part of our efforts in this direction, we are now requesting that all authors identified as 'corresponding author' create and link their Open Researcher and Contributor Identifier (ORCID) with their account on the Manuscript Tracking System prior to acceptance. ORCID helps the scientific community achieve unambiguous attribution of all scholarly contributions. You can create and link your ORCID from the home page of the Manuscript Tracking System by clicking on 'Modify my Springer Nature account' and following the instructions in the link below. Please also inform all co-authors that they can add their ORCIDs to their accounts and that they must do so prior to acceptance.
https://www.springernature.com/gp/researchers/orcid/orcid-for-nature-research

For more information please visit http://www.springernature.com/orcid

If you experience problems in linking your ORCID, please contact the Platform Support Helpdesk.

This email has been sent through the Springer Nature Tracking System NY-610A-NPG&MTS

Confidentiality Statement:

This e-mail is confidential and subject to copyright. Any unauthorised use or disclosure of its contents is prohibited. If you have received this email in error please notify our Manuscript Tracking System Helpdesk team at http://platformsupport.nature.com .

Details of the confidentiality and pre-publicity policy may be found here http://www.nature.com/authors/policies/confidentiality.html

Privacy Policy | Update Profile

DISCLAIMER: This e-mail is confidential and should not be used by anyone who is not the original intended recipient. If you have received this e-mail in error please inform the sender and delete it from your mailbox or any other storage mechanism. Springer Nature Limited does not accept liability for any statements made which are clearly the sender's own and not expressly made on behalf of Springer Nature Ltd or one of their agents.

Please note that Springer Nature Limited and their agents and affiliates do not accept any responsibility for viruses or malware that may be contained in this e-mail or its attachments and it is your responsibility to scan the e-mail and attachments (if any).

Springer Nature Ltd. Registered office: The Campus, 4 Crinan Street, London, N1 9XW. Registered Number: 00785998 England.

Reviewers' comments:

Reviewer #1 (Remarks to the Author):

The revised version addressed most of my comments, though I cannot say I'm fully satisfied with readability of the revised text. I places problems remain; some are listed below. These could be addressed in a minor revision.

Line 45: “Two basic mating systems, outbreeding and inbreeding… The former (dioecy or dioicy)… the latter (hermaphrodite, cosexuality, monoecy, or monoicy)” This is incorrect. Both inbreeding (mating with self, or close relatives) and outbreeding (mating with unrelated individuals) can occur in species with separate sexes and in cosexuals. Similarly, at line 58 it is incorrect to equate heterothallic to outbreeding and homothallic to inbreeding.

Line 65: “U (male) or V (female)”. Is it not the other way around? V-chromosome is usually male-specific and U is female-specific

Lines 90-91: “unique type of sex determination; elucidating its molecular and genomic bases will greatly improve our understanding of the diversity and evolution of mating systems” This kind of statements are usually found in grant proposals and it is annoying to see this in a research paper. Let the readers decide whether this paper will “greatly improve understanding” or not.

Line 94: Presumably the study was undertaken to understand the mating system rather than just “to generate whole genome sequences”?

Line 97: please replace “resolved exceptional” with “revealed unusual”. The same on line 328: replace “resolved very unique” with “revealed unusual”.

Line 135: “their SDR and PARs were identical, with three inverted regions” Confusing to see inverted regions mentioned after the statement that the regions are identical. Please split this sentence into the sentence about male-bisex comparison and another one about male-female comparison.

Line 139: Please start a new paragraph here. This would make the text better structured and will ensure the info about the genes is in a separate paragraph from the structural info described earlier. I also suggest to separate divergence analysis (from line 154) into a separate paragraph. This splitting will also make the structure of this subsection more balanced. Currently it looks odd that there is one very long paragraph + a very short one (about GC) at the very end.

Reviewer #2 (Remarks to the Author):

I found the manuscript to be substantially improved. Most of my comments for clarification or for further speculation have been addressed. Below I pinpoint places where I think that there is still room for improvement.

The introduction still lacks clarity. The opening of this section contains inaccurate statements such as « outbreeding … includes two separate sex phenotypes ». This is incorrect : many outbreeding species are actually hermaphroditic, thanks to e.g. self-incompatibility systems in flowering plants. I feel that the link between inbreeding/outbreeding and sex phenotypes, as it is currently exposed, introduces

unnecessary complications and could be greatly simplified.
The last sentence of the first paragraph is also unclear, and could be rewritten as « Trioecy is very rare across the tree of life, and the genetics of sex determination has been deciphered in a very small number of species only, including the nematode Auanema and the flowering plant Carica papaya » (just a suggestion).
In this first paragraph also, no need to spell « dioicy » and « monoicy ». Rather stick to just one notation.

The description of the sex-related genes in the introduction (lines 65-78) is now much better.

Line 76 : did you mean « paralogs » ?

line 87 : it is not clear what is meant by « the male and bisexual phenotypes have the identical « male SDR », while the female phenotype harbors the female SDR ». This gives the (false?) impression that the sequences of the SDRs had already been determined, while I understand that the previous study was based on crossing experiments only. Or are you talking about a previous genome assembly ? This paragraph should be improved to better highlight what was known already and the open questions that remained.

Line 113 : this is unclear : which particular statistics of the BUSCO analysis are you refering to ?

Line 144 : maybe briefly remind the reader why this is an « interesting » observation.

While I am not a native english speaker myself, I feel that the language still lacks clarity at many places of the manuscript, and many gramatical errors or misspelling remain.

Our responses to the reviewers' comments have been described below# in red.

Sincerely,

Dr. Hisayoshi Nozaki

#Reviewers' comments:

Reviewer #1 (Remarks to the Author):

The revised version addressed most of my comments, though I cannot say I'm fully satisfied with readability of the revised text. I places problems remain; some are listed below. These could be addressed in a minor revision.

Line 45: "Two basic mating systems, outbreeding and inbreeding… The former (dioecy or dioicy)… the latter (hermaphrodite, cosexuality, monoecy, or monoicy)" This is incorrect. Both inbreeding (mating with self, or close relatives) and outbreeding (mating with unrelated individuals) can occur in species with separate sexes and in cosexuals. Similarly, at line 58 it is incorrect to equate heterothallic to outbreeding and homothallic to inbreeding.

Response: Based on the comment, the sentences (line 45 and line 58) have been revised by deleting "outbreeding" and "inbreeding".

Line 65: "U (male) or V (female)". Is it not the other way around? V-chromosome is usually male-specific and U is female-specific

Response: Based on the comment, the revision has been done. In the Figure 1 legend, U or V chromosome had been already correctly described.

Lines 90-91: "unique type of sex determination; elucidating its molecular and genomic bases will greatly improve our understanding of the diversity and evolution of mating systems" This kind of statements are usually found in grant proposals and it is annoying to see this in a research paper. Let the readers decide whether this paper will "greatly improve understanding" or not.

Response: Based on the comments, this sentence (after the semicolon) has been deleted in the revised manuscript.

Line 94: Presumably the study was undertaken to understand the mating system rather than just "to generate whole genome sequences"?

Response: The sentence has been revised as suggested.

Line 97: please replace “resolved exceptional” with “revealed unusual”. The same on line 328: replace “resolved very unique” with “revealed unusual”.

Response: The two portions have been revised as suggested.

Line 135: “their SDR and PARs were identical, with three inverted regions” Confusing to see inverted regions mentioned after the statement that the regions are identical. Please split this sentence into the sentence about male-bisex comparison and another one about male-female comparison.

Response: The corresponding sentences have been revised as suggested.

Line 139: Please start a new paragraph here. This would make the text better structured and will ensure the info about the genes is in a separate paragraph from the structural info described earlier. I also suggest to separate divergence analysis (from line 154) into a separate paragraph. This splitting will also make the structure of this subsection more balanced. Currently it looks odd that there is one very long paragraph + a very short one (about GC) at the very end.

Response: Based on the comments, two new paragraphs have been started (lines 139 and line 154). In addition, the first paragraph of the Discussion section has been divided into two paragraphs in the revised manuscript.

Reviewer #2 (Remarks to the Author):

I found the manuscript to be substantially improved. Most of my comments for clarification or for further speculation have been addressed. Below I pinpoint places where I think that there is still room for improvement.

The introduction still lacks clarity. The opening of this section contains inaccurate statements such as « outbreeding … includes two separate sex phenotypes ». This is incorrect : many outbreeding species are actually hermaphroditic, thanks to e.g. self-incompatibility systems in flowering plants. I feel that the link between inbreeding/outbreeding and sex phenotypes, as it is currently exposed, introduces unnecessary complications and could be greatly simplified.

Response: Based also on the comment by the other reviewer, the Introduction section has been revised and re-examined by DeepL Write <https://www.deepl.com/write>.

The last sentence of the first paragraph is also unclear, and could be rewritten as « Trioecy is very rare across the tree of life, and the genetics of sex determination has been deciphered in a very small number of species only, including the nematode Auanema and the flowering plant Carica papaya » (just a suggestion).

Response: The sentence has been revised as suggested.

In this first paragraph also, no need to spell « dioicy » and « monoicy ». Rather stick to just one notation.

Response: « dioicy » and « monoicy » have been deleted as suggested.

The description of the sex-related genes in the introduction (lines 65-78) is now much better.

Response: Thank you!

Line 76 : did you mean « paralogs » ?

Response: For examples, *MTD1* are recognized in SDR or autosomal regions depending upon the volvocine species, but the gene phylogeny is consistent with the host phylogeny (Fig. 2C). Thus, these genes can be considered "orthologs." However, in order to avoid confusions for various readers, "orthologs" have been changed to "homologs" in this sentence and another (p. 6) in the revised manuscript.

line 87 : it is not clear what is meant by « the male and bisexual phenotypes have the identical « male SDR », while the female phenotype harbors the female SDR ». This gives the (false?) impression that the sequences of the SDRs had already been determined, while I understand that the previous study was based on crossing experiments only. Or are you talking about a previous genome assembly ? This paragraph should be improved to better highlight what was known already and the open questions that remained.

Response: Based on the suggestions, the paragraph and Figure 1 legend have been revised.

Line 113 : this is unclear : which particular statistics of the BUSCO analysis are you refering to ?

Response: Particular statistics of the BUSCO analysis had been described in the Materials and Methods section. Thus, "(see Materials and Methods)" has been described just after "(BUSCO) analysis" in the present revised manuscript.

Line 144 : maybe briefly remind the reader why this is an « interesting » observation.

Response: Based on the comments, the sentence has been revised.

While I am not a native english speaker myself, I feel that the language still lacks clarity at many places of the manuscript, and many gramatical errors or misspelling remain.

Response: The original manuscript had been revised by native English speakers/writers of Textcheck (The English in this document has been checked by at least two professional editors, both native speakers of English. For a certificate, please see: http://www.textcheck.com/certificate/HggXU0). In the revised portions of the text, we carefully re-examined the sentences by using DeepL Write <https://www.deepl.com/write>.
